# Cooperative ligand binding to a double-stranded Ising lattice—Application to cofilin binding to actin filaments

**DOI:** 10.1093/pnasnexus/pgad331

**Published:** 2023-10-11

**Authors:** Wenxiang Cao, Edwin W Taylor, Enrique M De La Cruz

**Affiliations:** Department of Molecular Biophysics and Biochemistry, Yale University, New Haven, CT 06520, USA; Department of Molecular Biophysics and Biochemistry, Yale University, New Haven, CT 06520, USA; Department of Molecular Biophysics and Biochemistry, Yale University, New Haven, CT 06520, USA

## Abstract

Cooperative ligand binding to linear polymers is fundamental in many scientific disciplines, particularly biological and chemical physics and engineering. Such ligand binding interactions have been widely modeled using infinite one-dimensional (1D) Ising models even in cases where the linear polymers are more complex (e.g. actin filaments and other double-stranded linear polymers). Here, we use sequence-generating and transfer matrix methods to obtain an analytical method for cooperative equilibrium ligand binding to double-stranded Ising lattices. We use this exact solution to evaluate binding properties and features and analyze experimental binding data of cooperative binding of the regulatory protein, cofilin, to actin filaments. This analysis, with additional experimental information about the observed bound cofilin cluster sizes and filament structure, reveals that a bound cofilin promotes cooperative binding to its longitudinal nearest-neighbors but has very modest effects on lateral nearest-neighbors. The bound cofilin cluster sizes calculated from the best fit parameters from the double-stranded model are considerably larger than when calculated with the 1D model, consistent with experimental observations made by electron microscopy and fluorescence imaging. The exact solution obtained and the method for using the solution developed here can be widely used for analysis of variety of multistranded lattice systems.

**Significance Statement**

Cooperative ligand binding to linear polymers is fundamental in many scientific disciplines. Such ligand binding has been widely modeled using 1D Ising models, due to the mathematical simplicity and analytical and numerical solvability. However, many biological polymers are double stranded. The additional dimension renders the model challenging to solve and practically implement for experimental data analysis. Here, we provide analytical solutions for cooperative equilibrium ligand binding to double-stranded lattices using sequence-generating and transfer matrix methods. We use the exact solution to simulate binding curves, and analyze experimental binding data of cofilin binding to actin filaments. The exact solution obtained and the method for using the solution developed here can be widely used for analysis of other double-stranded and multistranded systems.

## Introduction

The actin cytoskeleton is a dynamic network of semiflexible polymers that generates, responds, and adapts to mechanical forces in eukaryotic cells (1, 2). Filaments are noncovalent, linear polymers that elongate and shorten at filament ends through subunit addition and dissociation, respectively. Numerous fundamental biological processes, including cell division, growth, intracellular, and overall cell motility, rely on rapid growth and remodeling of the actin filament cytoskeleton.

Members of the ADF/cofilin family of filament regulatory proteins (herein referred to as cofilin) play essential roles in the assembly dynamics, reorganization, and force-generating properties of the actin cytoskeleton (3, 4). Cofilin severs actin filaments, which increases the concentration of filament ends from which polymer subunits add and dissociate (5, 6). Cofilin binds actin filaments with positive cooperativity (7–10), forming clusters of contiguously bound cofilin along filaments (7, 11–13). Filament severing occurs preferentially at junctions, or boundaries, between bare and cofilin-decorated cluster segments (3, 14–16). These cooperative binding interactions control the severing activity by influencing the cluster size and boundary density. Bound cofilin molecules do not directly interact, indicating that cooperative interactions are allosterically propagated through the filament lattice. Recent high-resolution, electron cryomicroscopy (cryo-EM) structures of actin-cofilactin boundaries (12) and of single, bound cofilin [i.e. “isolated”; (17)] indicate that filament structural perturbations by bound cofilin extend only 1–2 actin filament subunits, explaining why nearest-neighbor interactions account for cooperative cofilin binding to actin filaments (3, 4, 7).

Analytically, the equilibrium and kinetic cofilin binding to actin filaments has been modeled as a one-dimensional (1D) linear Ising model with nearest-neighbor binding interactions [i.e. only the adjacent neighbors on each side of a bound cofilin are affected (7, 8, 10, 14, 15, 18)] following principles derived for analysis of ligand binding to DNA (19). In this model, an actin filament is treated as a 1D lattice of identical binding sites at which of cofilin or other ligands can bind.

Three types of binding modes exist on a linear lattice (7, 8, 19, 20): (1) isolated (no bound nearest-neighbors) with an intrinsic association equilibrium constant (*K_a_*) for binding to an isolated site(s), (2) singly contiguous (one bound nearest-neighbor, on only one side) with a binding constant of *K_a_ω*, where *ω* is the cooperativity parameter (unitless), which reflects relative affinity for binding to a contiguous vs. an isolated site, and (3) doubly contiguous (two bound nearest-neighbors, one at each side) with affinity *K_a_ω*^2^, cooperativity contributed from both nearest-neighbors. Positive cooperativity (e.g. a bound ligand enhances binding of ligand to a neighboring site) is associated with an *ω* > 1; negative cooperativity (e.g. a bound ligand weakens binding of ligand to a neighboring site) generates *ω* < 1, and *ω* = 1 when no cooperative interactions exist. This nearest-neighbor model assumes cooperative interactions do not extend beyond an adjacent site.

The exact solutions for the binding of large ligands (binding stoichiometry *≥* 1 ligand per filament subunit or binding site) to a 1D lattice model with nearest-neighbor binding cooperativity has been solved by a variety of mathematical approaches such as transfer matrix (21–23), sequence generating (24), combinatorial method, and sequence generating (25), conditional probability (19), and site-specific thermodynamics and the properties of contracted partition functions (26), for example. Various quantitative analyses of cooperative cofilin equilibrium binding to actin filaments have been done by applying the general exact solution of the equilibrium ligand binding to a 1D lattice model with nearest-neighbor cooperative binding interactions (3, 4, 7, 8, 10, 14, 15, 27).

**Fig. 1.**
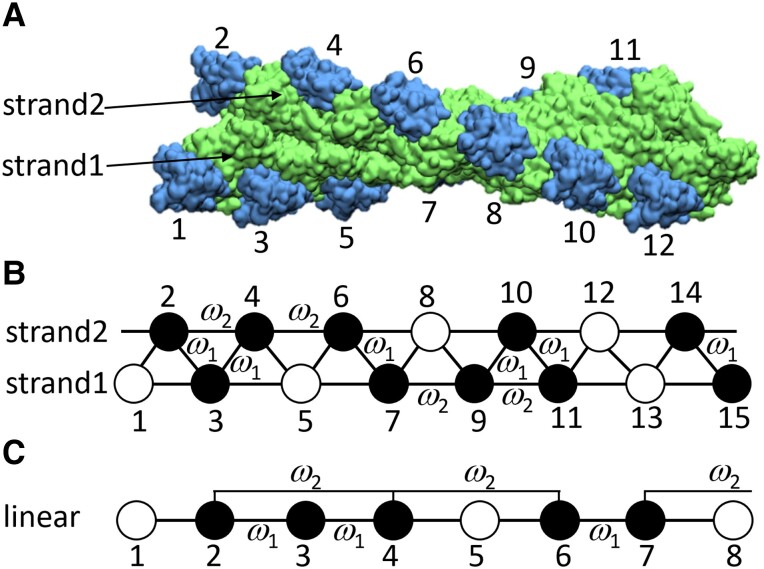
Bound ligands on a double helical lattice sites (double-stranded Ising model) contact and could potentially influence binding to the lateral and longitudinal neighbors. A) 9-Å resolution three-dimensional reconstruction of cofilin-decorated actin filament [PDB 3J0S (28)]. The actin filament is a double helical-stranded structure (lime color) and cofilin molecules (blue color) bind to filament subunits in both strands. B) A schematic of a double-stranded Ising model partially decorated with a ligand that binds stoichiometriccally (1 ligand:1 site; e.g. cofilin). Occupied (“bound”) sites are shown as filled circles and vacant (“free”) sites are represented by open circles. A bound ligand interacts with the nearest lateral (across) and longitudinal (horizontal) neighbors with cooperativity parameter *ω*_1_ and *ω*_2_, respectively. C) Mapping lattice sites 1–8 in a double-stranded lattice to a linearized 1 dimensional Ising lattice. In the mapping, the lateral (*ω*_1_) and longitudinal (*ω*_2_) cooperativities for interactions between the nearest-neighbor bound ligand pairs in double-stranded Ising lattice become cooperatives for interactions between the nearest-neighbor bound ligand pairs (*ω*_1_) and second nearest-neighbor bound ligand pairs (*ω*_2_) in the 1D lattice, respectively.

Structurally, an actin filament is not a 1D lattice. It resembles a topologically closed, 2D lattice, which has been described as either a double-stranded, right-handed, helical lattice comprised of two long-pitch helices, or protofilaments (referred to as a two-start, helix), or a single-stranded, left-handed, short-pitch helical lattice [referred to as a single-start, “genetic helix”; Fig. 1A, green molecules (28, 29)]. Protofilament stands have identical actin subunits and, hence, cofilin-binding sites (Fig. 1), and each cofilin-binding site has four nearest-neighbor binding sites—two longitudinal and two lateral. The geometry and cooperative interactions of lateral and longitudinal nearest-neighbors need not be identical. In fact, the interface areas and interaction energies of lateral and longitudinal filament interactions differ significantly (30–32).

A model of ligand binding to a double-stranded filament with asymmetric lateral and longitudinal cooperative interactions more precisely resembles the binding of ligands and regulatory proteins to actin filaments. A few studies have approached this problem using computational methods, including cooperative cofilin (33, 34) and tropomyosin (35) binding to actin filaments. To the best of our knowledge, an exact solution for cooperative ligand binding to a double-stranded lattice has not been provided or implemented for the data fitting and analysis of ligand binding to such polymers, including actin filaments.

Here, we derive an analytical method for cooperative ligand binding to double-stranded actin filaments. We use both sequence generating (main text) and transfer matrix (Online Supplementary Material) methods with identical results. The equilibrium binding isotherm curves generated from the exact solutions of both mathematical approaches are identical to simulated binding curves generated from Monte Carlo (MC) simulations. The sequence-generating method has an advantage over the transfer matrix approach because it can be easily formulated to relate the cluster size of a bound ligand to the total occupancy. We therefore present this method in the main text and present the transfer matrix approach in the Online Supplementary Material for comparison.

The bound ligand (e.g. cofilin) cluster size calculated with the double-stranded model developed here is significantly larger than that predicted by a 1D filament model (7), reconciling differences in experimental observations of the bound cofilin cluster size along actin filaments (12, 13, 17) and theoretical predictions (7). The method used in this work and solutions of this double-stranded lattice model can be applied to the analysis of ligand binding to other biological and synthetic multistranded polymers.

## Materials and methods

### Theory

#### Double-stranded Ising model and its linearization

Cofilin binds actin filaments stoichiometrically [1 cofilin per actin filament subunit (7, 28)] between longitudinally adjacent actin subunits (Fig. 1A, blue molecules). Accordingly, cofilin-binding sites along filaments can be modeled as two parallel, linear, 1D Ising lattice strands (Fig. 1B; cofilin-binding sites are numbered alternately across the two strands). In this double-stranded Ising model, cofilin binds the filament lattice at any site with an intrinsic binding affinity, association constant *K_a_* (dissociation binding constant *K_d_* = 1/*K_a_*). Filled circles represent cofilin-occupied sites (referred to as “bound”), while open circles represent unoccupied sites (referred to as “free”) available for cofilin binding. A bound cofilin may interact cooperatively with other cofilin molecules bound at laterally and/or longitudinally adjacent sites (Fig. 1B). There are therefore two distinct cooperativity parameters: one accounting for lateral interactions (*ω*_1_) and the other for longitudinal (*ω*_2_) interactions (Fig. 1B), such that the affinity for binding to a free site is given by *K_a_ω*_1_ or *K_a_ω*_2_ when an adjacent lateral or longitudinal site, respectively, has a bound cofilin.

To find an explicit mathematical solution to this double-stranded lattice model, we project laterally adjacent sites of the double-stranded lattice as linearly connected sites in a 1D lattice (see Fig. 1C). After this linearization, a longitudinal nearest-neighbor is represented as a second nearest-neighbor. This mapping is purely conceptual and not structural and retains the geometry of the double-stranded model despite being expressed in a 1D format. That is, linearization introduces no structural or geometric changes in the lattice sites and the longitudinal and lateral bonds between the lattice sites remain the same before and after linearization. We solved this linearized model analytically following two different approaches (sequence-generating and transfer matrix methods), yielding identical results.

#### Solution from the sequence-generating method

The grand partition function for the equilibrium binding of ligand to a lattice of sites is the sum of all possible microscopic states of the lattice with different ligand occupancies, where each individual site can exist as either unoccupied (free) or occupied. An individual microstate represents a given, unique pattern of bound ligand along a lattice. Macroscopically, this yields a lattice with alternating free and occupied ligand clusters (sequences) of different lengths. One approach to solve this partition function is to group all sites according to type (free or occupied) and summing over all possible clusters (sequences) according to each cluster's statistical weight. The grand partition function can then be directly solved from the two separate summations of all free and occupied site clusters. This approach is referred to as the sequence-generating method (36).

Following Shneior Lifson's pioneering work with the sequence-generating method (36), we built sequences of ligand bound and free lattice sites along our linearized model, allowing for cooperative binding interactions between nearest-neighbor and second nearest-neighbor pairs. Here, a “sequence” is a mathematical expression for the statistical weight of a cluster (i.e. a contiguous group) of either unoccupied sites (open circles, a sequence of free sites) or ligand-occupied sites (filled circles, a sequence of occupied sites).

We let *U* be a “sequence-generating function” of the free site cluster sequences. It is defined mathematically as the sum of all sequences (i.e. clusters) of unoccupied sites in an infinite lattice:


(1) $$\begin{array}{rl} U(\lambda ) & =\cdots ∙\circ ∙\cdots +\cdots ∙\circ \circ ∙\cdots +\cdots ∙\circ \circ \circ ∙\cdots +\cdots \\ & =e_{0}^{\prime }\omega_{2}\lambda^{-1}+c\sum_{i=2}^{\infty }{e_{0}}^{i}\lambda^{-i}=e_{0}^{\prime }\omega_{2}\lambda^{-1}+\frac{{ce_{0}}^{2}\lambda^{-2}}{1-e_{0}\lambda^{-1}}, \end{array}$$

where • is a ligand-occupied site (“bound”) and o is an unoccupied site (“free”). A cluster of contiguous sites, bound or free, with length *n* is comprised of *n* number of identical sites. Each site is associated with a partition function. In Eq. 1, *e′*_0_ is the partition function of an isolated, free site (*n* = 1), *e*_0_ is the partition function for unoccupied site clusters of *n* ≥ 2, *ω*_1_ is the cooperativity for a pair of bound, nearest-neighbor ligands (lateral neighbors in a double-stranded geometry), and *ω*_2_ is the cooperativity for a pair of next nearest-neighbor bound ligands (longitudinal neighbors in a double-stranded geometry).

The partition function of an unoccupied site sequence of length *n* = 1 (i.e. ⋯∙∘∙⋯ a free site with bound ligand at each side) is equal to the product of the vacant site partition function (*e′*_0_) and the cooperativity parameter of the second nearest-neighbor (i.e. longitudinally adjacent sites in 2D model: *i* ± 1) at both ends (*ω*_2_). The partition function of a free site sequence of length 2 is *e*_0_ × *e*_0_, and so on. The two bound ligands at both ends of an *n* ≥ 2 vacant site cluster (⋯∙∘∘∙⋯) are positioned beyond the second nearest-neighbor and do not interact, so do not contribute to the partition functions. The sequence for a *n*-cluster is defined as *λ*^−n^ times the partition function of the cluster, which is the product of all partition functions of all members and their interaction in the cluster. The term *λ* is a quantity whose value is to be solved, representing the contribution of each individual subunit to the grand partition function of the model *Ξ*^(*N*)^ (see below Eq. 5) when the lattice chain is sufficiently long and the end effect is negligible (36). The parameter *c* tags a sequence of empty site clusters with size ≥ 2 used by Schellman (24) to extract terms associated with those clusters. It has no physical meaning and is set to unity when analysis of cluster sizes is not needed (discussed below). We use it here for formulating a cluster size expression.

We let *V* be a “sequence-generating function” of the occupied site cluster sequences. For ligand-occupied sites in the lattice, the sum of all sequences (*V*) is given by:


(2) $$\begin{array}{rl} V(\lambda ) & =\cdots \circ ∙\circ \cdots +\cdots \circ ∙∙\circ \cdots +\cdots \circ ∙∙∙\circ \cdots +\cdots \\ & =x^{\prime }\lambda^{-1}+\omega_{1}x^{2}\lambda^{-2}+{\omega_{1}}^{2}\omega_{2}x^{3}\lambda^{-3}+{\omega_{1}}^{3}{\omega_{2}}^{2}x^{4}\lambda^{-4}+\ldots \\ & =x^{\prime }\lambda^{-1}+\frac{\omega_{1}x^{2}\lambda^{-2}}{1-\omega_{1}\omega_{2}x\lambda^{-1}}, \end{array}$$

where *x*′ is the partition function for an isolated-occupied site (*n* = 1; ⋯∘∙∘⋯), *x* is the partition function for the occupied sites in an *n* ≥ 2 cluster. Table 1 defines the partition functions associated with the different sites of a double-stranded lattice and equation parameters in all equations.

**Table 1.** Definition of parameters in equations and their values in our model.

*e′*_0_ = 1	partition function of an isolated free site
*e*_0_ = 1	partition function of a free site in an *n* ≥ 2 free site cluster
*x′* = *K_a_L* = *L*/*K_d_*	partition function of an isolated bound ligand
*x* = *K_a_L* = *L*/*K_d_*	partition function of a bound ligand in an *n* ≥ 2 contiguous ligand cluster
*ω*_1_	cooperativity parameter for lateral bound ligands on a double-stranded lattice partition function of pairwise interaction of a pair of first neighbors on a linearized double-stranded lattice
*ω*_2_	cooperativity parameter for longitudinal bound ligand interactions on a double-stranded lattice partition function of pairwise interaction of a pair of second neighbors on a linearized double-stranded lattice
*c* = 1	a marker for an *n* ≥ 2 free site cluster
*λ*	to be solved partition function of a subunit
*L_f_, L_t_*	free and total ligand (cofilin) concentration in solution
*K_a_*, *K_d_* = 1/*K_a_*	association and dissociation constant for binding to an isolated free site

For the infinite series in Eq. 1 to converge, *e*_0_/*λ* < 1 (i.e. *λ* > *e*_0_ = 1), and for the infinite series in Eq. 2 to converge, it must be *ω*_1_*ω*_2_*x*/*λ* < 1 (i.e. *λ* > *ω*_1_*ω*_2_*x*). Collectively, for both of these series to converge, *λ* > max(1, *ω*_1_*ω*_2_*x*); that is, *λ* must be larger than both 1 and *ω*_1_*ω*_2_*x*.

The characteristic equation for solving parameter *λ* is given by (36):


(3) $$U(\lambda )V(\lambda )-1=\left(\omega_{2}e_{0}^{\prime }\lambda^{-1}+\frac{ce_{0}^{2}\lambda^{-2}}{1-e_{0}\lambda^{-1}}\right)\left(x^{\prime }\lambda^{-1}+\frac{\omega_{1}x^{2}\lambda^{-2}}{1-\omega_{1}\omega_{2}x\lambda^{-1}}\right)-1=0,$$

which may be re-expressed as a fourth-order polynomial in the form:


(4) $$\begin{array}{rl} \lambda^{4} & -(e_{0}+\omega_{1}\omega_{2}x)\lambda^{3}-\omega_{2}(e_{0}^{\prime }x^{\prime }-e_{0}\omega_{1}x)\lambda^{2}\\ & -({ce_{0}}^{2}x^{\prime }+e_{0}^{\prime }\omega_{1}\omega_{2}x^{2}-e_{0}e_{0}^{\prime }\omega_{2}x^{\prime }-e_{0}^{\prime }\omega_{1}{\omega_{2}}^{2}x^{\prime }x)\lambda \\ & \;-e_{0}\omega_{1}x(e_{0}^{\prime }{\omega_{2}}^{2}x^{\prime }-ce_{0}\omega_{2}x^{\prime }-e_{0}^{\prime }\omega_{2}x+ce_{0}x)=0 \end{array}$$

with four roots. When the total number of lattice binding sites *N* is sufficiently large, the grand partition function of the model becomes (36):


(5) $$\Xi^{\left(N\right)}∼{\lambda_{1}}^{N},$$

where *λ*_1_ is the largest of the four positive roots of Eq. 4 (36). Only the largest root (*λ*_1_) of Eq. 4 must be solved for determining the ligand concentration-dependence of the lattice occupancy (36).

The partition function (statistical weight; *x′* or *x*) for an individual-occupied site is proportional to the free ligand concentration (*L_f_*) and ligand equilibrium association binding constant (*K_a_*), i.e. *x′* = *x* = *K_a_L_f_* = *L_f_*/*K_d_* (Table 1). Therefore, the differential of the grand partition function of the double-stranded lattice model (Eq. 5) with respect to *x* is the average number of bound ligands (*N*_bound_) (24):


(6) $${\bar{N}}_{\mathrm{bound}}=\frac{x}{\Xi^{\left(N\right)}}\frac{d\Xi^{\left(N\right)}}{dx}$$

from which the binding density (*ν*) can be calculated according to:


(7) $$v=\frac{{\bar{N}}_{\mathrm{bound}}}{N}=\frac{1}{N}\frac{x}{\Xi^{\left(N\right)}}\frac{d\Xi^{\left(N\right)}}{dx}=\frac{1}{N}\frac{x}{{\lambda_{1}}^{N}}\frac{{d\lambda_{1}}^{N}}{dx}=\frac{x}{\lambda_{1}}\frac{d\lambda_{1}}{dx},$$

where *N* is the total number of lattice binding sites.

Most cofilin-binding parameters (4), including the stoichiometry (7, 28), are known (Fig. 1A; Table 1), and their values are given by:


(8) $$e_{0}^{\prime }=e_{0}=1,x^{\prime }=x=K_{a}L_{f}=L_{f}/K_{d},\;\mathrm{and}\;c=1,$$

where *L_f_* is the free ligand concentration. Substituting these known parameters into Eq. 4 yields:


(9) $$\begin{array}{rl} \lambda^{4} & -(1+\omega_{1}\omega_{2}x)\lambda^{3}+\omega_{2}x(\omega_{1}-1)\lambda^{2}+x(\omega_{2}-1)(1+\omega_{1}\omega_{2}x)\lambda \\ & -\omega_{1}x^{2}(\omega_{2}-1{)}^{2}=0, \end{array}$$

which is one the characteristic equation to be solved in data analysis and simulation with the double-stranded model. Using Eq. 7, it can be proven that the largest root, *λ*_1_ ≥ 1 (see Online Supplementary Material).

When there is no cooperativity between longitudinal neighbors (e.g. longitudinally adjacent neighbor in the explicit double-stranded lattice model and the second nearest-neighbor in the linearized form of the double-stranded model; Fig. 1B and C), *ω*_2_ = 1. In this case, the double-stranded model reduces to a 1D lattice model with only nearest-neighbor cooperative interactions, and Eq. 9 simplifies to:


(10) $$\begin{array}{rl} & \lambda^{2}-(1+\omega_{1}x)\lambda +x(\omega_{1}-1)=\lambda^{2}-\lambda -x(\omega_{1}(\lambda -1)+1)=0;\\ & x=\lambda \frac{\lambda -1}{\omega_{1}(\lambda -1)+1}. \end{array}$$

Equation 10 is the characteristic equation for 1D nearest cooperative binding model derived by the sequence-generating function (37) and transfer matrix (23, 38) methods. In the Online Supplementary Material, we provide a proof showing that the larger root (*λ*_+_) in this case is always ≥ 1, whereas the smaller root (*λ*_−_) is always 0 ≤ *λ*_−_ ≤ 1, thus providing boundary conditions when running simulations and fitting data.

To derive a binding density equation for the model, we differentiate both sides of Eq. 9 with respect to *x* and substitute $\frac{d\lambda_{1}}{dx}$ from Eq. 7, yielding after rearranging terms:


(11) $$\frac{v}{x}=\frac{1}{\lambda_{1}}\frac{\omega_{1}\omega_{2}{\lambda_{1}}^{3}-\omega_{2}(\omega_{1}-1){\lambda_{1}}^{2}-(\omega_{2}-1)(1+2\omega_{1}\omega_{2}x)\lambda_{1}+2x\omega_{1}{\left(\omega_{2}-1\right)}^{2}}{4{\lambda_{1}}^{3}-3(1+\omega_{1}\omega_{2}x){\lambda_{1}}^{2}+2\omega_{2}x(\omega_{1}-1)\lambda_{1}+x(\omega_{2}-1)(1+\omega_{1}\omega_{2}x)},$$

where *x* = *K_a_L_f_*. Equation 11 is a general exact solution of our double-stranded ligand binding lattice model. It is a general binding formula for calculating the ligand concentration-dependence of the ligand binding density (*ν*) at thermodynamic equilibrium that can be used for fitting experimental data and simulating binding curves when combined with the largest root obtained with Eq. 9.

As noted above, when *ω*_2_ = 1, the double-stranded model reduces to a 1D lattice model. Combining Eqs. 10 and 11 yields the Scatchard form of the equation historically used for presenting cooperative ligand binding data [e.g. (19, 23), see Online Supplementary Material]:


(12) $$\frac{v}{x}=(1-v){\left(\frac{1-2v+R}{2(1-v)}\right)}^{2},$$

where


(13) $$R=\sqrt{{\left(1-2v\right)}^{2}+4\omega_{1}v(1-v)}.$$

#### Average number and length of bound and free site clusters

A bound ligand cluster in a 1D lattice is defined as a group of contiguously bound lattice sites with no intermittent free sites (referred to as “gaps”; e.g. ligands bound at sites 2, 3, and 4 in Fig. 1C). The same applies for free site clusters. Thus, there is no ambiguity on what defines a contiguous cluster on a 1D lattice.

In contrast, a bound ligand cluster in a double-stranded lattice must be explicitly defined, since a contiguous cluster exists even when there is an empty site gap within the bound cluster. For example, ligands bound at sites 2–4, 6–7, and 9–11 in Fig. 1B comprise a contiguous cluster despite there being single site gaps at positions 5 and 8. This is because the bound ligands occupying both sides of the gaps are adjacent to and contact their longitudinal nearest-neighbors (Fig. 1B) and thus part of a contiguous cluster. In other words, single site gaps on a double-stranded lattice are not true gaps and *≥* 2 contiguous free sites are needed to break a contiguous cluster. Accordingly, a contiguous cluster on a double-stranded lattice represents a composite of contiguous bound and isolated free sites, and its length is given by the total number of these sites in the cluster. In the corresponding linearized model (Fig. 1C), this is illustrated as a second nearest-neighbor contact across a single empty site gap. Below, we distinguish between the two classes of contiguously bound clusters—those accommodating single site gaps and those that do not (i.e. clusters that treat single site gaps as breaks of a contiguous cluster).

We note that when only two types of clusters can exist (e.g. bound and free), the two cluster types alternate along the lattice. Therefore, the numbers of bound and free clusters in a finite lattice differ by no more than ±1 and are equal on an infinite lattice. This is true for clusters that allow single-site gaps as well as for those that do not.

To obtain the average length of bound ligand clusters, we first determine the total number of single free site gaps ($n_{\mathrm{gap}=1}$) and the number of gaps with length *≥* 2, which can be calculated from the grand partition function of the system (discussed in detail below), and hence from the largest root of characteristic function according to Eq. 5. In general, characteristic equation (Eq. 4), a gap of length = 1 (i.e. a single isolated free site) is marked by the parameter *e*′_0_, while a free site gap of length *≥* 2 is marked by the parameter *c*. Since the largest root of Eq. 4 for *λ* is an implicit function of those parameters, the derivatives of the largest root *λ*_1_ to parameters *e*′_0_ and *c* can be carried out by differentiating both sides of Eq. 4 with respect to *e*′_0_ and *c*. After rearranging terms, those derivatives become:


(14) $$e_{0}^{\prime }\frac{\partial \lambda }{\partial e_{0}^{\prime }}=\frac{e_{0}^{\prime }\omega_{2}(x^{\prime }\lambda^{2}+(\omega_{1}x^{2}-e_{0}x^{\prime }-\omega_{1}\omega_{2}x^{\prime }x)\lambda +e_{0}\omega_{1}x(\omega_{2}x^{\prime }-x))}{4\lambda^{3}-3(e_{0}+\omega_{1}\omega_{2}x)\lambda^{2}-2\omega_{2}(e_{0}^{\prime }x^{\prime }-e_{0}\omega_{1}x)\lambda -{ce_{0}}^{2}x^{\prime }-e_{0}^{\prime }\omega_{1}\omega_{2}x^{2}+e_{0}e_{0}^{\prime }\omega_{2}x^{\prime }+e_{0}^{\prime }\omega_{1}{\omega_{2}}^{2}x^{\prime }x},$$

(15) $$c\frac{\partial \lambda }{\partial c}=\frac{{ce_{0}}^{2}(x^{\prime }\lambda +\omega_{1}x(-\omega_{2}x^{\prime }+x))}{4\lambda^{3}-3(e_{0}+\omega_{1}\omega_{2}x)\lambda^{2}-2\omega_{2}(e_{0}^{\prime }x^{\prime }-e_{0}\omega_{1}x)\lambda -{ce_{0}}^{2}x^{\prime }-e_{0}^{\prime }\omega_{1}\omega_{2}x^{2}+e_{0}e_{0}^{\prime }\omega_{2}x^{\prime }+e_{0}^{\prime }\omega_{1}{\omega_{2}}^{2}x^{\prime }x}.$$

Following Schellman's method (24) and using the partition function in Eq. 5 and derivatives in Eqs. 14 and 15, the total number of single site gap (${\bar{n}}_{\mathrm{gap}=1}$) and number of gaps with length > 2 (${\bar{n}}_{\mathrm{gap}\geq 2}$) can be expressed and derived as follows:


(16) $$\begin{array}{rl} {\bar{n}}_{\mathrm{gap}=1} & ={\frac{d\ln\;\Xi^{\left(N\right)}}{d\ln\;e_{0}^{\prime }}|}_{e_{0}^{\prime }=1}={\frac{e_{0}^{\prime }N}{\lambda_{1}}\frac{d\lambda_{1}}{de_{0}^{\prime }}|}_{e_{0}^{\prime }=1}\\ & =\frac{N}{\lambda_{1}}\frac{\omega_{2}x(\lambda^{2}-((\omega_{2}-1)\omega_{1}x+1)\lambda_{1}+\omega_{1}x(\omega_{2}-1))}{4{\lambda_{1}}^{3}-3(1+\omega_{1}\omega_{2}x){\lambda_{1}}^{2}+2\omega_{2}x(\omega_{1}-1)\lambda_{1}+x(1+\omega_{1}\omega_{2}x)(\omega_{2}-1)} \end{array}$$

(17) $$\begin{array}{rl} {\bar{n}}_{\mathrm{gap}\geq 2} & ={\frac{d\ln\;\Xi^{\left(N\right)}}{d\ln\;c}|}_{c=1}={\frac{cN}{\lambda_{1}}\frac{d\lambda_{1}}{dc}|}_{c=1}\\ & =\frac{Nx}{\lambda_{1}}\frac{\lambda_{1}-\omega_{1}x(\omega_{2}-1)}{4{\lambda_{1}}^{3}-3(1+\omega_{1}\omega_{2}x){\lambda_{1}}^{2}+2\omega_{2}x(\omega_{1}-1)\lambda_{1}+x(\omega_{2}-1)(1+\omega_{1}\omega_{2}x)}, \end{array}$$

where *N* denotes the number of lattice sites (*N* = *N*_occupied_ + *N*_unoccupied_) and *n* denotes the number of clusters (bound or free). Note that the number of isolated bound or free sites is equal to the number of the clusters (i.e. *N*_single_ = *n*_single_).

Substituting the parameter values given by Eq. 8 into Eqs. 16 and 17, then using Eqs. 16 and 17 and the binding density (Eq. 11), the average bound ligand cluster size including those with free single site gaps (${\bar{C}}_{\mathrm{sgap}}$) can be expressed as follows:


(18) $$\begin{array}{rl} {\bar{C}}_{\mathrm{sgap}} & =\frac{{\bar{N}}_{\mathrm{bound}}+{\bar{N}}_{\mathrm{gap}=1}}{{\bar{n}}_{\mathrm{gap}\geq 2}}=\frac{vN+{\bar{n}}_{\mathrm{gap}=1}}{{\bar{n}}_{\mathrm{gap}\geq 2}}\\ & =\frac{\omega_{1}\omega_{2}{\lambda_{1}}^{3}+\omega_{2}(2-\omega_{1}){\lambda_{1}}^{2}-((\omega_{2}-1)(1+3\omega_{1}\omega_{2}x)+\omega_{2})\lambda_{1}+(3\omega_{2}-2)\omega_{1}x(\omega_{2}-1)}{\lambda_{1}-\omega_{1}x(\omega_{2}-1)}. \end{array}$$

For comparison, the average size of contiguously bound ligands in clusters that *do not* allow single site gaps (i.e. a single free site is treated as a break in the cluster) is given by (${\bar{C}}_{\mathrm{nogap}}$):


(19) $$\begin{array}{rl} {\bar{C}}_{\mathrm{nogap}} & =\frac{{\bar{N}}_{\mathrm{bound}}}{{\bar{n}}_{\mathrm{gap}}}=\frac{\nu N}{{\bar{n}}_{\mathrm{gap}\geq 2}+{\bar{n}}_{\mathrm{gap}=1}}\\ & =\frac{\omega_{1}\omega_{2}{\lambda_{1}}^{3}-\omega_{2}(\omega_{1}-1){\lambda_{1}}^{2}-(\omega_{2}-1)(1+2\omega_{1}\omega_{2}x)\lambda_{1}+2x\omega_{1}{\left(\omega_{2}-1\right)}^{2}}{\omega_{2}{\lambda_{1}}^{2}-(\omega_{2}\omega_{1}x+1)(\omega_{2}-1)\lambda_{1}+\omega_{1}x{\left(\omega_{2}-1\right)}^{2}}. \end{array}$$

When *ω*_2_ = 1, according to Eqs. 18 and 19 and the *λ*_1_ express in Eq. S8 in Online Supplementary Material, the average size of bound ligand clusters with and without single site gaps are given by (see Eqs. S25 and S26 in Online Supplementary Material):


(20) $${\bar{C}}_{\mathrm{sgap}}=\frac{\omega_{1}(1+R)-2}{(2\omega_{1}-1)(1-v)+v-R},$$

(21) $${\bar{C}}_{\mathrm{nogap}}=2\frac{v(\omega_{1}-1)}{\left(R-1\right)}=\frac{R+1}{2(1-\nu )},$$

where *R* is expressed in Eq. 13. We note that the expression in Eq. 21 (Online Supplementary Eq. S26) is identical to that previously derived with the conditional probability method for ligand binding to 1D lattice with nearest-neighbor interactions and a binding stoichiometry of 1 (39). In Eq. S34 (Online Supplementary Material), we also prove Eq. 20 (Online Supplementary Eq. S25) is identical to that derived with the conditional probability method for ligand binding to 1D lattice.

The (average) total number of free sites is given by *N*(1 − *v*) and the (average) total number of free sites in gaps with length ≥2 is given by $N(1-v)-{\bar{n}}_{\mathrm{gap}=1}$. Accordingly, the average length of free site gaps with length ≥2 (${\bar{G}}_{\mathrm{gap}\geq 2}$) is given by:


(22) $$\begin{array}{rl} {\bar{G}}_{\mathrm{gap}\geq 2} & =\frac{{\bar{N}}_{\mathrm{free}}-{\bar{N}}_{\mathrm{gap}=1}}{{\bar{n}}_{\mathrm{gap}\geq 2}}=\frac{N(1-v)-{\bar{n}}_{\mathrm{gap}=1}}{{\bar{n}}_{\mathrm{gap}\geq 2}}\\ & =\frac{\begin{array}{c} 4{\lambda_{1}}^{4}-(3+4\omega_{1}\omega_{2}x){\lambda_{1}}^{3}+\omega_{2}x(3\omega_{1}-4){\lambda_{1}}^{2}+x(2(1+2\omega_{1}\omega_{2}x)(\omega_{2}-1)+\omega_{2})\lambda_{1}\\ -\omega_{1}x^{2}(3\omega_{2}-2)(\omega_{2}-1) \end{array}}{x(\lambda_{1}-\omega_{1}x(\omega_{2}-1))}. \end{array}$$

Similarly, the average length of free site gaps including single site gaps (${\bar{G}}_{\mathrm{all}}$) is given by:


(23) $$\begin{array}{rl} {\bar{G}}_{\mathrm{all}} & =\frac{{\bar{N}}_{\mathrm{free}}}{{\bar{n}}_{\mathrm{gap}\geq 2}+{\bar{n}}_{\mathrm{sgap}}}=\frac{N(1-v)}{{\bar{n}}_{\mathrm{gap}\geq 2}+{\bar{n}}_{\mathrm{sgap}}}\\ & =\frac{4{\lambda_{1}}^{4}-(3+4\omega_{1}\omega_{2}x){\lambda_{1}}^{3}+3\omega_{2}x(\omega_{1}-1){\lambda_{1}}^{2}+x(\omega_{2}-1)(2+3\omega_{1}\omega_{2}x)\lambda_{1}-2x^{2}\omega_{1}{\left(\omega_{2}-1\right)}^{2}}{x(\omega_{2}{\lambda_{1}}^{2}-(1+\omega_{2}\omega_{1}x)(\omega_{2}-1)\lambda_{1}+\omega_{1}x{\left(\omega_{2}-1\right)}^{2})}. \end{array}$$

Note that in the absence of ligand (i.e. *x* = 0), the free site gap lengths ${\bar{G}}_{\mathrm{all}}$ and ${\bar{G}}_{\mathrm{gap}\geq 2}\to \infty$.

#### Solution from transfer matrix method approach

In the Online Supplementary Material, we use the transfer matrix method to derive characteristic equation (Eq. S46) in a different form than shown in Eq. 9, as well as the binding equation (Eq. S48) in a different form than shown in Eq. 11. However, the binding curves generated from both methods overlay (Online Supplementary Fig. S1), indicating that despite different analytic expressions, the solutions for the ligand binding density obtained by both approaches are consistent (discussed below). The sequence-generating method has the advantage over the transfer matrix method in that it readily allows for calculation of the cluster sizes. The derivations are also simpler with the sequence-generating method, as are the analytical expressions for solutions.

### Equilibrium binding titrations

#### Proteins

Rabbit skeletal muscle actin was purified from the leg and back muscle of freshly sacrificed rabbits and purified as described (40), labeled with *N*-pyrenyl iodoacetamide (pyrene), and gel-filtered over Sephacryl S300 at 4°C in buffer A (2 mM Tris-Cl (pH 8.0 at 25°C), 0.2 mM K^+^ ATP, 0.5 mM DTT, 0.2 mM CaCl_2_, 1 mM NaN_3_) (7, 8). Recombinant human nonmuscle cofilin-1 was expressed in *Escherichia coli* and purified as described (41). Pyrene-labeled Ca^2+^-actin monomers were converted to Mg^2+^-actin monomers with 0.2 mM EGTA and 50 μM MgCl_2_ immediately before polymerizing by dialysis against KMI_6.6_ buffer [50 mM KCl, 0.2 mM ATP, 2 mM MgCl_2_, 2 mM DTT, 1 mM NaN_3_, 20 mM imidazole (fluorescence grade) pH 6.6] (7, 8).

#### Equilibrium binding titrations of cofilin and actin filaments

Cofilin binding quenches the fluorescence of pyrene-labeled actin, such that the observed fluorescence signal is proportional to the cofilin occupancy (7). A broad concentration range of cofilin was mixed with 1.8 μM pyrene-actin and equilibrated at room temperature for ≥ 2 h. The fluorescence intensity of equilibrated samples was measured with a Photon Technology International, Inc. (South Brunswick, NJ) Quanta master fluorimeter at 25°C. Samples were excited at 366 nm and the fluorescence emission at 90° was scanned from 390 to 600 nm. The observed pyrene fluorescence intensities at 407 nm (peak) were plotted against total cofilin concentration and fitted with procedures described below. The obtained binding constant and cooperativities from data fitting were averaged according to the standard deviation from the fits as weighting (weighted average). The data was normalized to the best fit parameters for presentation.

### Simulation and analysis

#### Simulation of equilibrium ligand binding titration curves and cluster size determinations

A custom program written in both Python (with NumPy/SciPy) and Matlab (www.mathworks.com) was used to simulate binding curves as a function of the free ligand concentration (*L_f_*) in either the conventional (*ν* vs. *L_f_*) or Scatchard (*ν*/*L_f_* vs. *ν*) plot formats. Two different procedures were followed, with identical results. In one procedure, the free ligand concentration, *L_f_* (i.e. *x* = *K_a_L_f_* = *L_f_*/*K_d_*), is an independent variable ranging from 0 to +∞ and the fourth-order polynomial equation (Eq. 9) is solved numerically for the largest root *λ*_1_ followed by calculating the binding density (*ν*) from Eq. 11. In the second procedure, the parameter *λ* (Eq. 9) serves as the independent parameter and is confined to *λ ≥* 1 (for it to be the largest root of Eq. 9; see the proof in the Online Supplementary Material) after which *x* (i.e. *L_f_*) is calculated from the only physically meaningful root of the quadratic equation of *x* (Eq. 9; see Eq. S17 in Online Supplementary Material):


(24) $$x=\frac{2{\lambda_{1}}^{2}(\lambda_{1}-1)}{(\omega_{1}\lambda_{1}+1)\omega_{2}(\lambda_{1}-1)+1+\sqrt{{\left((\omega_{1}\lambda_{1}+1)\omega_{2}(\lambda_{1}-1)+1\right)}^{2}-4\omega_{1}(\omega_{2}-1)(\omega_{2}(\lambda_{1}-1)+1)\lambda_{1}(\lambda_{1}-1)}}$$

and the binding density (*ν*) is calculated from Eq. 11. With the generated *ν*, *x*, and *λ*, the bound ligand and gap cluster sizes can be calculated with Eqs. 18, 19, 22, and 23.

#### Fitting equilibrium binding titration curves

A custom program used for fitting simulated and experimental data to the double-stranded model was written with Origin C, based on C program language, using software Origin (originlab.com). When experimental data is expressed as a function of *L_f_*, a procedure similar to simulations described above can be used to generate *λ* and *ν* from *x* (i.e. numerically solved Eq. 9 for the largest root, *λ*_1_, and substituted *λ*_1_, *x* into Eq. 11 to calculate *ν*).

When the free ligand concentration is not known and binding curves are plotted as a function of the total ligand concentration *L_t_* (*x* = *K_a_*(*L_t_*− *A_t_ν*), where *A_t_* are the total lattice site concentration, the fitting procedure is more complicated. To solve *λ*, it is convenient to define an implicit function (*f*) of *λ* (Eq. 25) from mass conservation and its derivative to *λ* (Eq. 26) for solving *λ* numerically from *f*(*λ*) = 0 at a given *L_t_*:


(25) $$f(\lambda )=L_{f}+A_{t}v-L_{t}=K_{d}x(\lambda )+A_{t}v(x,\lambda )-L_{t}=0,$$

(26) $$\begin{array}{rl} \frac{df(\lambda )}{d\lambda } & =K_{d}\frac{dx(\lambda )}{d\lambda }+A_{t}\left(\frac{\partial v(x,\lambda )}{\partial \lambda }+\frac{\partial v(x,\lambda )}{\partial x}\frac{dx(\lambda )}{d\lambda }\right)\\ & =\left(K_{d}+A_{t}\frac{\partial v(x,\lambda )}{\partial x}\right)\frac{dx(\lambda )}{d\lambda }+A_{t}\frac{\partial v(x,\lambda )}{\partial \lambda }. \end{array}$$

Following an iteration procedure to solve *λ* from the above equations, *x* and *ν* were calculated with Eqs. 24 and 11, respectively, and derivatives of $\frac{dx(\lambda )}{d\lambda }$, $\frac{\partial v(x,\lambda )}{\partial \lambda }$, and $\frac{\partial v(x,\lambda )}{\partial x}$ were calculated from their mathematical expressions by taking derivatives of Eq. 24 and both sides of Eq. 11 (see Eqs. S20–S22 in Online Supplementary Material). To ensure the value of *λ* represents the largest root of Eq. 9, it must be in the region *λ* ≥ 1 (Online Supplementary Material, first section). This fitting procedure was also used to directly simulate equilibrium binding titration curves as function of total ligand concentration *L_t_* without calculating free ligand concentration *L_f_* (*x*) first (i.e. generate *ν* at a given set of *L_t_* and parameters). The Matlab *m*-*file* implemented for these simulations is included as part of the Online Supplementary Material.

Similar to the procedures above, a custom program used for fitting simulated and experimental data as function of either *L_f_* or *L_t_* to the 1D Ising lattice model solved by McGhee and von Hippel (7, 8, 19) was also written with Origin C in software Origin (originlab.com; see Online Supplementary Material for details).

Fitting to the simulated data without noise (Fig. 4 and Fig. 6A, lower curve) was done without weighting, whereas fitting to data with noise (simulated, Fig. 6A, upper curve; or experimental Fig. 6B) was done with inverse of the signal value (1/signal, since the signal should follow Poisson distribution; Poisson variance = Poisson expectation = signal) as a weighting factor in the chi-square minimization procedure of the Levenberg-Marquardt least-squares curve fitting. The binding constant and cooperativities obtained from the best fits of the data represent (standard deviation) weighted averages. A text file with Origin C codes used in the curve fitting in software Origin (originlab.com) is also included as part of the Online Supplementary Material.

#### Simulation of ligand binding kinetics

A custom MC program written with Matlab scripts was used to simulate time courses of ligand binding to a double-stranded lattice of sites. The methods used for the simulations were based on published MC procedures (42, 43), following Epstein's algorithm (42) and the multistep binding kinetics treatment used by De La Cruz and Sept (18), but with an important modification. When calculating ligand binding at each time step, each event in the event queue was assigned as either an association or dissociation event by a random permutation shuffling procedure, rather than an ordered procedure [e.g. association events followed by dissociation (42)]. This modification eliminates inaccuracies caused by large time steps (see Fig. S2 in Online Supplementary Material for comparison).

We used this method to simulate the time courses of ligand binding to our double-stranded Ising lattice model until reaching the equilibrium (∼5 s) and then compared the binding density at equilibrium to that calculated from the exact solution of the model obtained by the sequence-generating method. MC simulations were done using a solution volume of 10 fL. The double-stranded lattice was linearized and modeled as a single continuous array (Fig. 1C), which corresponds to ∼9,000 total binding sites at a concentration of 1.5 μM sites (equivalent to [actin] in binding experiments). The number of ligand molecules varied from to 590 to ∼300,000, corresponding to a concentration range of 0.098 to 50 μM (equivalent to [cofilin] in binding experiments). The Matlab scripts for this program (MC_coop_ligand_bind_double_stranded.m) and additional details regarding the simulations are included in the Online Supplementary Material.

## Results and discussion

### Ligand binding isotherms

**Fig. 2.**
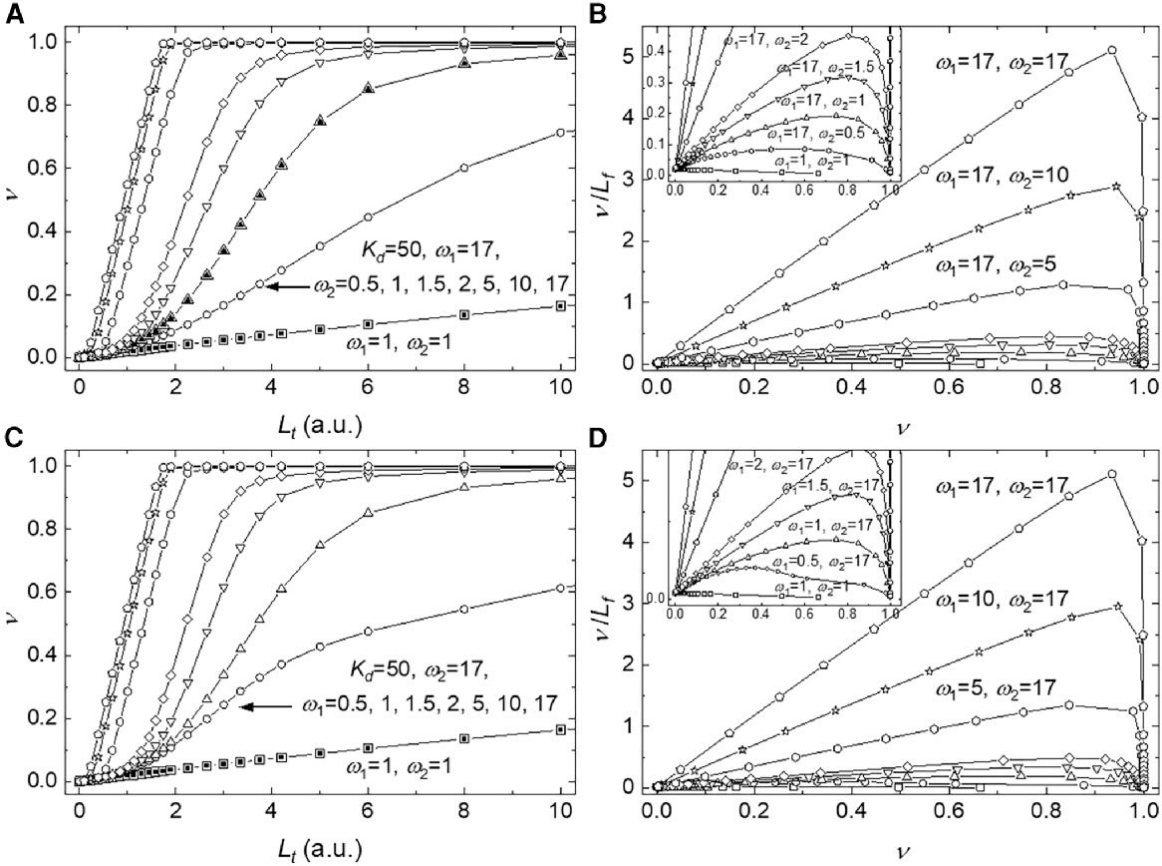
Simulation of ligand equilibrium binding titrations using the double-stranded lattice model solved by sequence-generating method. A and C) *ω*_1_ (A) or *ω*_2_ (C) dependence of binding curves as a function of the total ligand concentration were generated from the solutions by the sequence-generating method with Eqs. 9, 11, 25, and 26 (Materials and methods section). For all curves, the ligand binding density*ν* and the total ligand concentration *L_t_* vary, but the total lattice site concentration *A_t_* = 1.5 and the binding constant *K_d_* = 50 are fixed. The units for quantities *L_t_*, *A_t_*, and *K_d_* are arbitrary (a.u.) but matching; since all curves are simulated, the units can be arbitrarily defined provided they match. The lowest simulated curve in both Panels A and C has no binding cooperativity, and the rest of the curves all have *ω*_1_ (A) or *ω*_2_ (C) = 17, but *ω*_2_ (A) or *ω*_1_ (C) = 0.5, 1, 1.5, 2, 5, 10, and 17 (from right to left). B and D) Scatchard plot of the same data shown in Panels A and C, respectively. The inserts in Panels B and D show a close-up of the curves with lower *ω*_2_ (A) or *ω*_1_ (C).

**Fig. 3.**
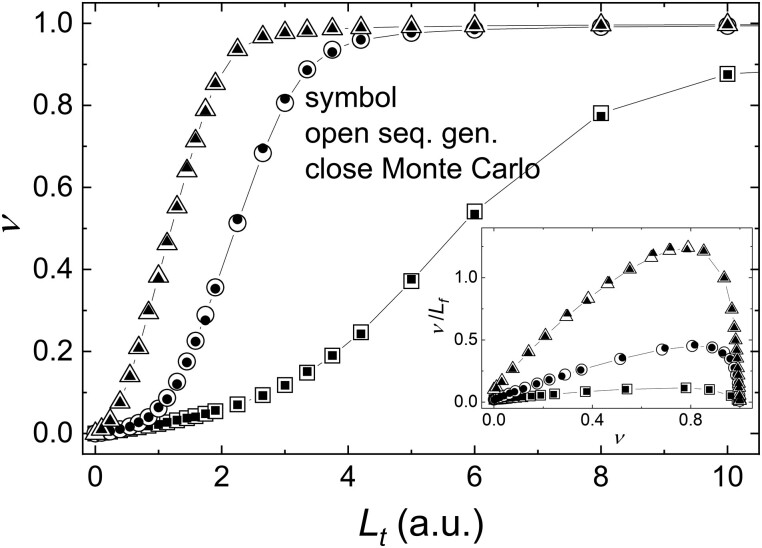
The simulated equilibrium binding titrations from the deterministic solutions solved by sequence-generating (open symbols) and Monte Carlo (closed symbols) methods overlay. All simulations were carried out with *A_t_* = 1.5. *K_d_* = 50, and *ω*_1_ and *ω*_2_ values are: *ω*_1_ = 2 and *ω*_2_ = 5 (squares) or *ω*_1_ = 17 and *ω*_2_ = 2 (circles). *K_d_* = 10, *ω*_1_ = 10 and *ω*_2_ = 2 (triangles). The inset shows the same data plotted in the Scatchard format. In the stochastic Monte Carlo simulations, the kinetic ligand binding data (closed symbols) were simulated until equilibrium was achieved (5 s) and the presented equilibrium binding density data represent the average of 11 data points from 4.8 to 5 s. All units are arbitrary, but matching.

Positive cooperative binding interactions makes curves of the ligand concentration-dependence of the binding density sigmoidal (rather than quadratic or hyperbolic) when plotted on a linear scale (Fig. 2A and C). When plotted in the Scatchard format, curves deviate from linearity (Fig. 2B and D). Binding curves also deviate from quadratic or hyperbolic when negative cooperative binding interactions exist (*ω* < 1; Fig. 2). The binding density from both stochastic and deterministic approaches overlay at every initial free ligand concentration (Fig. 3), independently verifying the exact solution derived here and used for fitting of experimental data.

### Cooperativity exchange symmetry

**Fig. 4.**
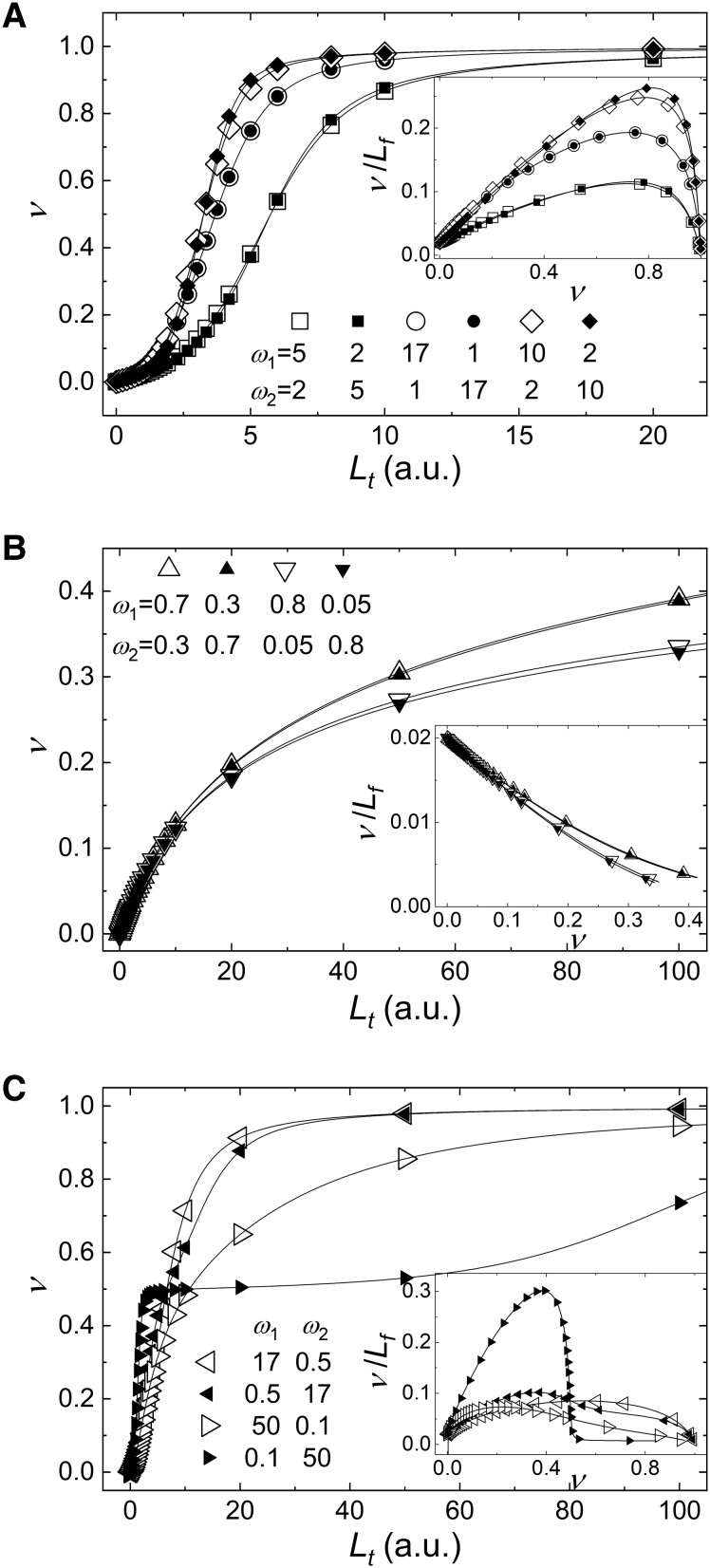
Binding curves have an approximate *ω*_1_ and *ω*_2_ exchange symmetry in some cases. All curves were simulated by sequence-generating method with *A_t_* = 1.5, *K_d_* = 50, and *ω*_1_ and *ω*_2_ values shown in plots. A and B) (negative) pairs of curves with *ω*_1_ and *ω*_2_ values exchanged approximately overlay when both cooperativities are either positive (including neutral; A) or negative (B). Differences are seen when both *ω*_1_ and *ω*_2_ are not equal to 1 (i.e. binding is cooperative). A pair of curves with *ω*_1_ and *ω*_2_ values swapped are plotted with the same symbol, but one is open and the other is filled. C) Curves with one cooperativity positive (e.g. *ω*_1_ = 17 or 50) and the other negative (e.g. *ω*_2_ = 0.5 or 0.1) exchanged do not overlay well. The continuous lines through the data in all plots represent the best fits to the same model and the recovered *K_d_*, *ω*_1_, and *ω*_2_ values by the fits match the values used in simulations within the standard deviations that are in the order of 10^−5^ smaller than the values reported by the fits. The insets in all panels show the same data plotted in Scatchard format.

A given change in the positive cooperativity of *ω*_1_ with no change in *ω*_2_ yields similar behavior as a corresponding change in the positive cooperativity of *ω*_2_ with no change in *ω*_1_ (Fig. 4A). That is, longitudinal and lateral cooperative interactions are interchangeable and display an approximate exchange symmetry. This exchange symmetry also holds when *ω*_1_ and *ω*_2_ both are ≤1 (i.e. no cooperativity or negative cooperativity; Fig. 4B). This exchange symmetry indicates that the two cooperativities (*ω*_1_ and *ω*_2_) equally affect ligand occupancy.

In contrast, when *ω*_1_ and *ω*_2_ cooperative interactions are opposite (i.e. one displaying positive cooperativity and the other displaying negative cooperativity), the exchange symmetry is lost, such that the binding curves do not overlay when *ω*_1_ and *ω*_2_ values are exchanged (Fig. 4C). For example, when *ω*_1_ displays negative cooperativity (*ω*_1_ < 1) and *ω*_2_ displays positive cooperativity (*ω*_2_ > 1), filaments achieve half saturation at low ligand concentrations (along one strand) but full saturation is difficult to achieve (filled, right-faced triangles in Fig. 4C), whereas full saturation is readily achieved when *ω*_1_ > 1 and *ω*_2_ < 1 (filled, left-faced triangles in Fig. 4C). Such asymmetric behaviors arise because longitudinal interactions (*ω*_2_) modulate growth along only one of the two strands, whereas lateral interactions (*ω*_1_) modulate interactions along both.

### Bound ligand and empty site clusters

**Fig. 5.**
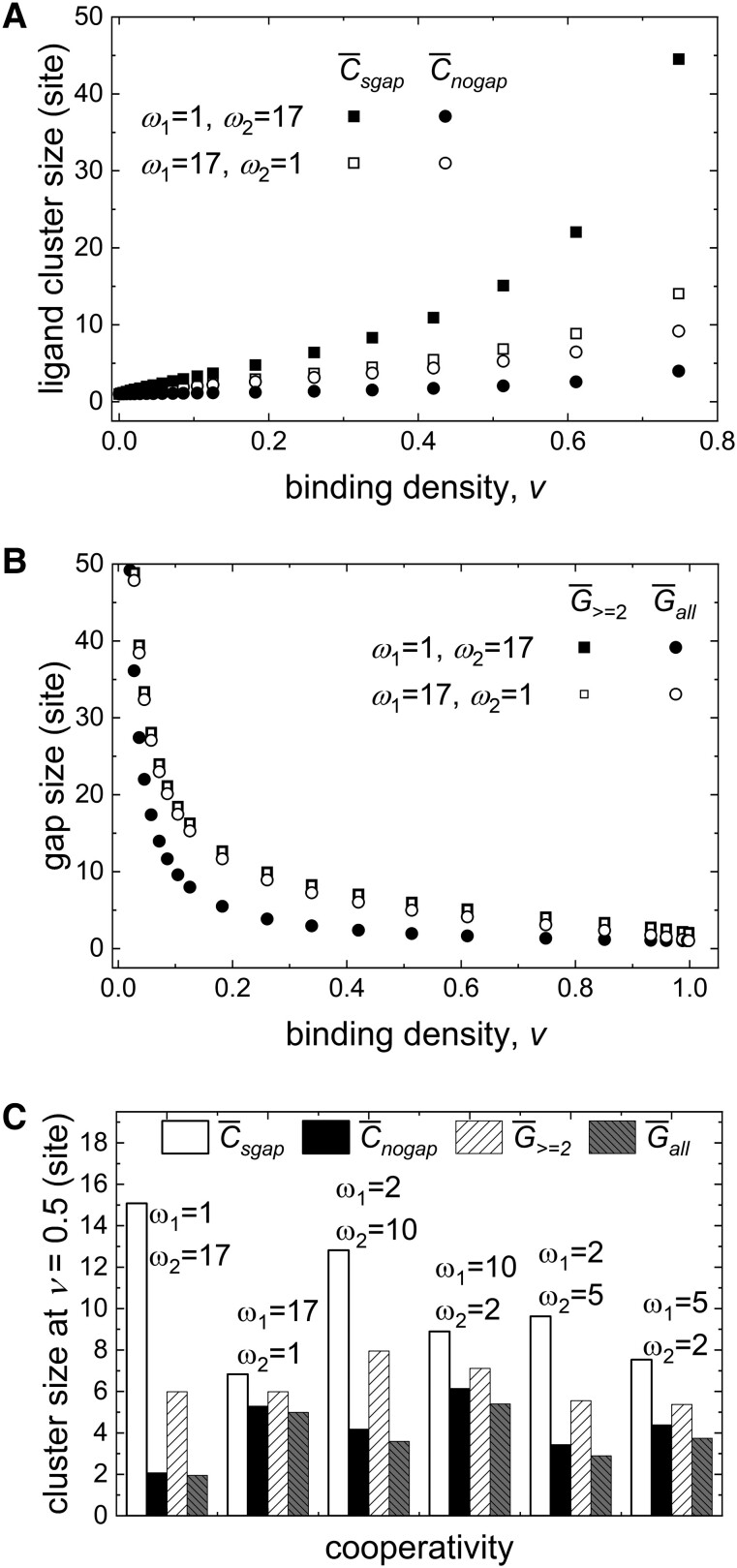
Positive longitudinal cooperativity produces more isolated single gaps than lateral cooperativity. A) Bound ligand cluster size comparison between single gaps allowed (${\bar{C}}_{\mathrm{sgap}}$; squares) and disallowed (${\bar{C}}_{\mathrm{nogap}}$, circles). B) Gap size comparison between excluding (${\bar{G}}_{\geq 2}$; squares) and including (${\bar{G}}_{\mathrm{all}}$; circles) single gaps. C) Comparison of average bound ligand cluster and gap sizes at *ν*∼ 0.5 for different cooperativity (*ω*_1_ and *ω*_2_) combinations.

The cluster sizes of occupied and empty sites can be directly calculated from the largest root *λ*_1_ and free ligand concentration *x* according to Eqs. 18, 19, 22, and 23 (Fig. 5). Both lateral (*ω*_1_) and longitudinal (*ω*_2_) cooperativities promote bound ligand cluster sizes (Fig. 5A) and lower the gap sizes (Fig. 5B). At binding densities *ν* > 0.1, the longitudinal cooperativity makes the average bound ligand cluster size much larger if single empty site gaps are included (Fig. 5A, filled symbols), whereas the lateral cooperativity has little effect on the average bound ligand cluster size, whether single empty site gaps are allowed or not (Fig. 5A, open symbols). This observation indicates that longitudinal cooperativity, but not lateral cooperativity, generates many isolated, single empty site gaps, which increase the average bound ligand cluster size when single gaps are allowed and decreases the cluster size when single gaps are forbidden. This phenomenon is supported by the gap size analysis (Fig. 5B). Since *ω*_2_ cooperativity creates many single site gaps and *ω*_1_ does not, the average gaps size becomes much shorter if those gaps are treated as such (Fig. 5B, filled symbols), while the average gap size does not change much whether single site gaps are included or not (Fig. 5, open symbols). The average bound ligand cluster and empty site clusters at half saturation (binding density *ν* = 0.5) further supports the conclusion that strong, positive longitudinal cooperativity (*ω*_2_ >> 1) generates more isolated single site gaps than strong, positive lateral cooperativity (*ω*_1_ >> 1; Fig. 5C).

When longitudinal cooperativity dominates, bound ligand cluster formation is favored along a single strand. This introduces single site gaps in bound clusters along the double-stranded lattice (i.e. filament). In contrast, when lateral cooperative interactions dominate, ligand binding across strands is favored and this precludes single site gap formation along the filament.

#### Analysis of equilibrium cofilin-binding data with the double-stranded lattice model

**Fig. 6.**
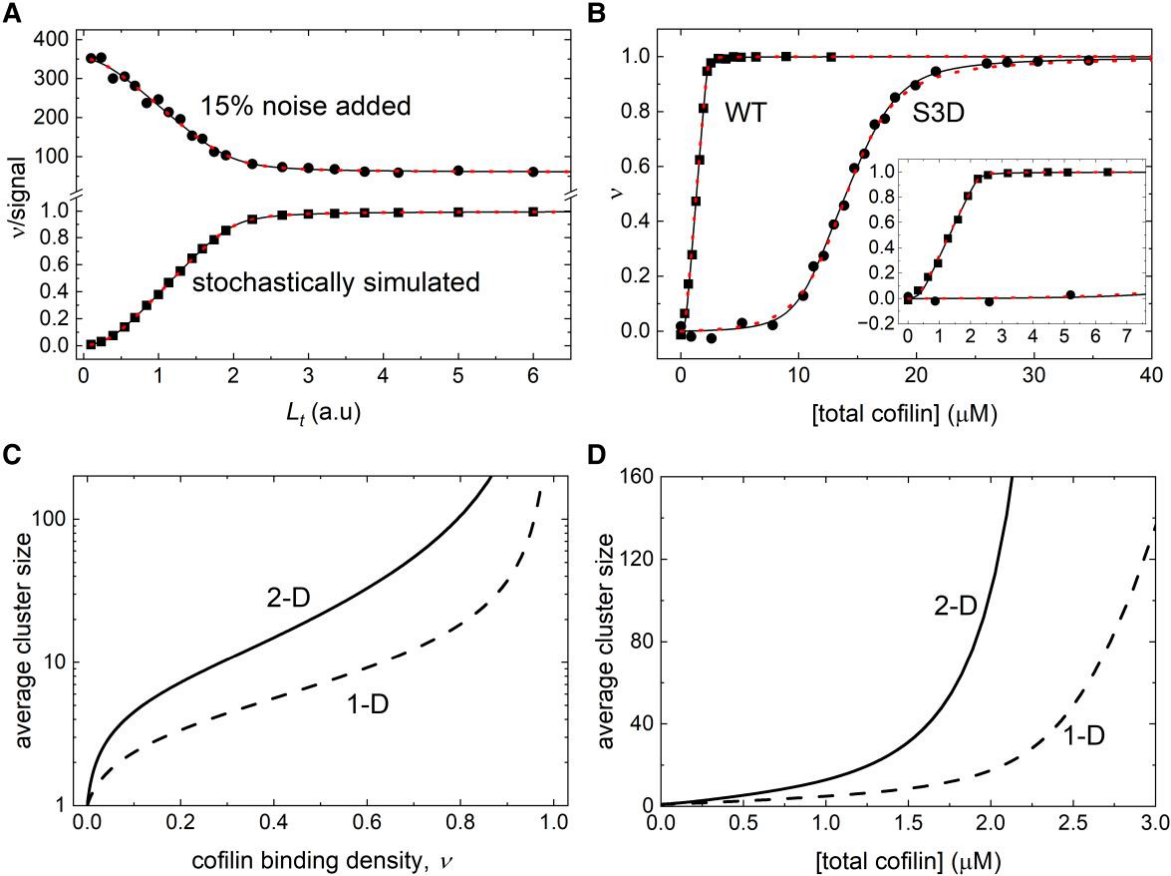
Equilibrium binding of cofilin and actin filaments analyzed with a double-stranded Ising model. A) Effects of random noise on data fitting. The lower binding curve (filled squares) was generated by the Monte Carlo simulation of kinetic ligand binding to the double-stranded Ising lattice model at equilibrium (*t* = 5 s) with *A_t_* = 1.4, *K_d_* = 10, *ω*_1_ = 10 and *ω*_2_ = 2. The upper binding curve (filled circles) was a representative of a set of simulated experimental data (15% random noise added) to mimic experimental signal (quenching) by bound ligand, created from the lower simulated curve by flipping, shifting and re-scaling arbitrarily, and finally supplementing with 5–15% noise to signal of uniform random noise [signal = ((1−simulated) × scale + background) × (1 + 0.15 × (uniform random noise−0.5))]. The continuous black lines through the data are the fits to the analytic solution of the double-stranded model (Eqs. 9 and 11) through a numerical procedure, yielding *K_d_* = 9.7 (±1.3), *ω*_1_ = 9.7 (±3.3), and *ω*_2_ = 2.0 (±0.4) for Monte Carlo simulated (lower filled squares) and *K_d_* = 9.3 (±1.5), *ω*_1_ = 8.9 (±4.2), and *ω*_2_ = 1. 2 (±0.3) the weighted average of the fits to five sets (*n* = 5) of the same simulated data but with different random noise (one set of 5%, one set of 10%, and three sets of 15%), background signal and scaling. The dotted red lines through the data are the fits to the 1D lattice model (19) for comparison, yielding *K_d_* = 16.6 (±1.0), *ω* = 33.6 (±1.8) for Monte Carlo simulated and *K_d_* = 14.7 (±1.4), *ω* = 30.4 (±2.6) the weighted average of the fits to five sets (*n* = 5) of the noise added data. B) Fitting and analysis of experimental data. The observed equilibrium binding density of WT (filled squares) and the phosphomimetic S3D mutant (filled circles) cofilin binding to actin filaments (*A_t_* = 1.8 μM for both experiments) are plotted as a function of the total cofilin concentration. The inset shows the same data over a narrower range. The continuous black lines though the data represent the best fits to the double-stranded model, yielding *K_d_* = 13.1 (±6.5) μM, *ω*_1_ = 0.89 (±0.03), and *ω*_2_ = 36.2 (±21.6) weighted average of the fits of seven sets of WT cofilin data and *K_d_* = 846 (±448) μM, *ω*_1_ = 0.92 (±0.03), and *ω*_2_ = 68 (±40) weighted average of the fits of two sets of S3D mutant cofilin data. The dotted red lines through the data represent the fits to the 1D lattice model, yielding *K_d_* = 9.5 (±2.9) μM, *ω* = 22.0 (±6.0) weighted average of the fits of seven sets of WT cofilin data and *K_d_* = 720 (±94) μM, *ω* = 54.4 (±7.0) weighted average of the fits of two sets of S3D mutant cofilin data. Fitting any data with noise (A, upper curve; B, experimental data) was done with weighting (see Materials and methods section). C) The cofilin binding density-dependence of the average cluster size. Clusters with single gaps were calculated according to the best fit parameters of the experimental data in Panel B, analyzed by 2D and 1D models. At a cofilin-binding density *ν* = 0.5, *C*_sgap_ = 21.1 (2D; Table 1) and 7.1 (1D; Table 1). D) The total [cofilin]-dependence of the average cluster size is shown in Panel C.

To test the accuracy and robustness of the analyses of experimental data with our exact solution, we simulated equilibrium ligand binding curves to the double-stranded Ising model using the stochastic MC method (Fig. 6A) or the exact solution (Eqs. 9 and 11, Fig. 4), and then fitted those simulated binding curves to the exact solution of the model (Materials and methods section, Figs. 4 and 6A). The fitting curve precisely overlays the simulated binding curve (Fig. 6A, lower curve and Fig. 4, all curves) and the unconstrained fitting parameter values of *K_d_*, *ω*_1_, and *ω*_2_ obtained match the values used to generate the simulated data (within 3%: Fig. 6A, simulated stochastically, or 10^−5^%: Fig. 4, simulated by the exact solution of relative deviation). The fitting curve generated from our exact solution of the model still overlays the simulated data (Fig. 6A, upper curve is a representative) when arbitrarily shifting and scaling the data and supplementing the simulated data with 5–15% noise-to-signal uniform random noise, but with larger relative deviation.

Experimental data of the equilibrium binding of cofilin and actin filaments are well fitted to the exact solution of the double-stranded model (Fig. 6B). The best unconstrained fit parameters of multiple titration data sets [*n* = 7 for wild-type (WT) and *n* = 2 for S3D mutant] consistently converge to longitudinal cooperative interactions (*ω*_2_) that are large and positive for both cofilin WT (*ω*_2_ ∼ 36, weighted average) and the S3D mutant (*ω*_2_ ∼ 68, weighted average), but lateral cooperative interactions (*ω*_1_) are very modest or negligible (*ω*_1_ ∼ 0.9 for both, weighted average). This cooperative binding behavior is consistent with the observed effects of cofilin occupancy on actin filament structure, which shows that a bound cofilin alters the structure of longitudinal, but not the lateral, neighboring binding site in a manner to favor cofilin binding (17, 33, 44, 45), without any long-range, nonnearest-neighbor structural changes. The higher cooperativity of S3D cofilin is also consistent with previous biochemical studies (14).

We note, however, that due to the exchange symmetry between lateral (*ω*_1_) and longitudinal (*ω*_2_) cooperativities when both are positive (≥1) or negative (≤1; previous section), the data are fitted equally well when *ω*_1_ and *ω*_2_ values are exchanged (Fig. 4 and Online Supplementary Fig. S3). Therefore, analysis of binding titration curves alone is not sufficient to properly assign the *ω*_1_ and *ω*_2_ values, and additional information is needed to assign the origins of the two cooperativities with confidence. The two *ω*_1_ and *ω*_2_ exchanged scenarios yield different cluster size distributions and the experimentally observed cluster size distributions (17) can thus help to properly assign *ω*_1_ and *ω*_2_ values when compared to experimental data.

The average contiguously bound cofilin cluster size at half saturation (i.e. binding density *ν* = 0.5) calculated using the 1D model is ∼4 cofilin molecules (7), considerably smaller than observed experimentally by cryo-EM, which estimated an average contiguous cluster size of ∼20 cofilin molecules (12, 17). Fluorescence imaging of cofilin-decorated filaments also observed clusters larger than predicted by the 1D model (13). When *ω*_2_ cooperativity is large and positive and *ω*_1_ ∼ 1, the average cluster sizes with single empty site gaps calculated with the double-stranded model are much larger than that with the 1D model, particularly at binding densities *ν* > 0.5 (Fig. 6C and D), accounting for the experimentally observed large cluster size for cooperative cofilin binding to actin filaments. When *ω*_1_ cooperativity is large and positive and *ω*_2_ ∼ 1, the double-stranded model reduces to the 1D model, and yields the same smaller cluster sizes, in contrast to what has been observed experimentally. Therefore, the bound cofilin cluster sizes observed by electron microscopy (12, 17) and by fluorescence microscopy of labeled cofilin and actin (13) favor a cofilin-binding mechanism in which longitudinal cooperative interactions (*ω*_2_) are large and positive and lateral cooperative interactions (*ω*_1_) are modest.

We note that this result (no lateral cooperative binding interactions) was unexpected, as cofilin has been shown to alter the filament structure in a manner that compromises both longitudinal and lateral interactions (44, 45). Our analysis indicates that the effects of the lateral interactions have modest effects, if any, on cooperative cofilin binding, though they may affect filament stability (15, 34, 46), mechanics (27, 47–49), and interactions with other ligands (50).

We also note that the intrinsic binding affinities for binding to an isolated site (*K_d_*) are similar when analyzed with 1D and double-stranded models (Table 2). The cooperativity (*ω*) determined with the 1D model compares to the value of the longitudinal cooperativity (*ω*_2_) determined with the double-stranded model. This is expected because longitudinal interactions dictate cooperative cofilin binding to actin (Table 2).

**Table 2.** Cofilin-binding constants and cluster sizes determined from experimental data fitting.

	WT cofilin (7 sets)		S3D cofilin (2 sets)	
	2D^a^	1D^b^	2D^a^	1D^b^
Weighted average of unconstrained fitting parameters				
* K_d_* (isolated; μM)	13.1 (±6.5)	9.5 (±2.9)	846 (±448)	720 (±94)
* ω*_1_^a^ (lateral)	0.89 (±0.03)	—	0.92 (±0.03)	—
* ω*_2_^a^ (longitudinal)	36.2 (±21.6)	—	68 (±40)	—
* ω* ^b^	—	22.0 (±6.0)	—	54.4 (±7.0)
Calculated according to fitting parameters				
* C*_sgap_ at *ν* = 0.5	21.1	7.1	29.1	9.6
* C*_nogap_ at *ν* = 0.5	1.8	5.7	1.7	8.4

^a^Fitting by Eqs. 9 and 11. ^b^Fitting by 1D Ising model, see Materials and methods section.

#### Applicability to other multistranded polymer lattices

The analysis method developed and presented here for cofilin and actin binding can be applied to other ligands and systems, including other proteins that bind actin filaments and other multistranded polymers. For example, binding of the muscle and nonmuscle cell regulatory protein, tropomyosin to actin filaments is highly cooperative, with binding to an adjacent site being favored by >1,000 times than an isolated site (51). It has long been established that direct longitudinal interactions between bound tropomyosin molecules promote cooperative binding to longitudinal sites (“end-to-end” cooperativity). However, recent binding and high-resolution, fluorescence imaging studies implicate lateral cooperative interactions (i.e. across strands) and long-range (“indirect” cooperativity) interactions as well (35). Similarly, binding and structural studies of muscle and nonmuscle myosin motor proteins have shown that binding to actin filaments is cooperative [e.g. (52–54), and references therein]. The regulatory proteins comprising the thin filament of muscle (i.e. tropomyosin and troponins) also promote cooperative myosin binding to actin filaments [e.g. (55)].

The approach presented here can be extended to other multistranded cytoskeleton polymers, such as microtubules. Various ligands bind microtubules and regulate their biological functions. Many of these bind cooperatively (56), including the medicinal drugs colchicine (to treat gout and other inflammations), vinblastine (chemotherapy), and maytansine (breast cancer treatment). Similarly, members of the kinesin superfamily of microtubule-based molecular motor proteins bind microtubules cooperatively (57, 58).

The analysis method presented here can also be applied to nonprotein polymer lattices that are comprised of multiple strands. For example, amylose adopts two distinct double-helical forms (A and B), as do nonnatural amylose analog polysaccharides (59). Amylose cooperatively binds aromatic molecules and fatty acids and that are widely used as fragrances and antibacterial agents in the food and cosmetic industry (60).

We anticipate that the analysis method presented here are of broad interest and will motivate and facilitate analysis of ligand binding and other (e.g. folding) interactions in natural and synthetic multi stranded polymer lattice systems.

#### Comparison to the exact solution of the model obtained by the transfer matrix method

To solve an Ising lattice model for an exact solution, the matrix transfer method is one of earliest and widely used methods (21–23, 61). We also solved the model by this method (Online Supplementary Material). The ligand binding formula (Online Supplementary Eq. S48) and the characteristic equation (Online Supplementary Eq. S46) do not resemble the ones solved by the sequence-generating method (Eqs. 11 and 9). Nevertheless, the binding curves simulated from the exact solutions of the model by the transfer matrix and by the sequence generating are on top each other (Online Supplementary Fig. S2), indicating the exact solutions from both methods are consistent with one another.

## Supplementary Material

pgad331_Supplementary_Data

## Data Availability

Source codes in Matlab *m*-file format for simulating equilibrium ligand binding to double-stranded lattice model with the nearest-neighbor cooperativities by the solution solved with the sequence-generating method and by the MC method were attached as parts of the Online Supplementary Material. Source code in Origin C (combination of C and C++ used in the software Origin coding) in plain text format and its compiled program used as Origin fitting program (*.fdf) for fitting data to our solution solved with the sequence-generating method were attached as parts of the Online Supplementary Material. All data is included in the manuscript and/or Online Supplementary Material.
