# Supplementary material for: Cooperative ligand binding to a double-stranded Ising lattice—Application to cofilin binding to actin filaments
Source: PNAS Nexus. 2023 Oct 11;2(10):pgad331. doi: 10.1093/pnasnexus/pgad331 (PMC10599439; doi:10.1093/pnasnexus/pgad331)
Supplement: pgad331_Supplementary_Data [file pgad331_supplementary_data.zip › PNASNEXUS-PNASNEXUS-2023-00886-T-s01.pdf]

Cooperative ligand binding to a double-stranded filament lattice – application to actin filaments

Wenxiang Cao, Edwin W. Taylor and Enrique M. De La Cruz

Department of Molecular Biophysics and Biochemistry, Yale University, New Haven, CT 06520, USA

Supplementary Information

1. *Proof that the largest root of characteristic equation Eq. 9 is* $\lambda_1 \geq 1$.

For the proof. we let $f(\lambda)$ equal to the left side of Eq. 9 as a function of λ:

$$f(\lambda) = \lambda^4 - (1+\omega_1\omega_2 x)\lambda^3 + \omega_2 x(\omega_1 - 1)\lambda^2 + x(\omega_2 - 1)(1+\omega_1\omega_2 x)\lambda - \omega_1 x^2(\omega_2 - 1)^2 \qquad \text{S1.}$$

The roots of polynomial Eq.9, λ_i, i = 1, …4, is the 0 points of function, i.e., satisfy $f(\lambda) = 0$. $f(\lambda)$ changes its sign as λ continuously changes while crossing a root. The only root of interest is the largest and also positive (1)

Since $x = K_a L_f$, x is proportional to the free ligand concentration L_f and must be $x \geq 0$. At $x = 0$, Eq. 9 simplifies to Eq. S2:

$$f(\lambda) = \lambda^4 - \lambda^3 = (\lambda - 1)\lambda^3 = 0 \qquad \text{S2}$$

yielding the largest root $\lambda_1 = 1$ (the only other root, a multiple (triplet) root, is $\lambda = 0$).

When $x > 0$, $\omega_2 \neq 1$ and $\lambda \to \pm\infty$, $f(\lambda) \to \lambda^4 > 0$, and at $\lambda = 0$, $f(0) = -\omega_1 x^2(\omega_2 - 1)^2 < 0$, i.e., $f(\lambda)$ has a different sign at $\lambda = 0$ from $\lambda \to \pm\infty$, indicating $f(\lambda)$ has at least a positive and a negative root. According to the sequence generating method (1) , only the largest positive root is the root of interest.

Since $x > 0$, $\lambda_1 > 0$, and $\nu > 0$, from Eq. 7, the derivative of λ_1 as function of x satisfies Eq. S3:

$$\frac{d\lambda_1}{dx} = \frac{\nu\lambda_1}{x} > 0 \qquad \text{S3.}$$

This means that for $x > 0$, the physically meaningful largest root of Eq. 9, λ_1 monotonically increases with x. Therefore, the minimum value of $\lambda_1 = 1$ is when $x = 0$, and $x > 0$, $\lambda_1 > 1$.

When $\omega_2 = 1$, the larger of the 2 roots in Eq. 10 is

$$\lambda_+ = \frac{1+\omega_1 x+\sqrt{(1+\omega_1 x)^2-4x(\omega_1-1)}}{2} \ge \frac{1+\omega_1 x+\sqrt{(1-\omega_1 x)^2}}{2} = \begin{cases} \omega_1 x \ge 1 & \text{if } \omega_1 x \ge 1 \\ 1 & \text{if } 0 \le \omega_1 x < 1 \end{cases}$$

and the smaller root is

$$\lambda_- = \frac{1+\omega_1 x-\sqrt{(1-\omega_1 x)^2+4x}}{2} \le \frac{1+\omega_1 x-\sqrt{(1-\omega_1 x)^2}}{2} = \begin{cases} 1 & \text{if } \omega_1 x \ge 1 \\ \omega_1 x < 1 & \text{if } 0 \le \omega_1 x < 1 \end{cases}$$

The larger root in this case is always $\lambda_+ \ge 1$, whereas the smaller root is always $0 \le \lambda_- \le 1$.

2. *Special cases of ligand binding density and characteristic equation derived using the Sequence generating method.*

When $x \to 0$, the largest root $\lambda_1 = 1$ (Eq. S2) and binding density equation (Eq. 11) simplifies to

$$\frac{\nu}{x} \xrightarrow{x=0,\lambda=1} \frac{\omega_1\omega_2-\omega_2(\omega_1-1)-(\omega_2-1)}{4-3} = \omega_1\omega_2-\omega_1\omega_2+\omega_2-\omega_2+1=1 \qquad \text{S4.}$$

Accordingly, the y-intercept in Scatchard plot, $\frac{\nu}{L_f} = K_a = \frac{1}{K_d}$ at $L_f = 0$ since $x = K_a L_f$,.

When $\omega_2 = 1$ (i.e., there is no the longitudinal binding cooperativity in a double stranded Ising model or no second nearest-neighbor binding cooperativity in the linearized Ising model), the characteristic equation (Eq. 9) becomes:

$$\lambda^4-(1+\omega_1 x)\lambda^3+x(\omega_1-1)\lambda^2 = \lambda^2\left(\lambda^2-(1+\omega_1 x)\lambda+x(\omega_1-1)\right)=0 \ .$$

There are 2 zero and 2 non-zero roots in the equation, and since the largest root has to be > 0, proved in Section 1 here, it must be

$$x = \frac{\lambda(\lambda-1)}{\omega_1(\lambda-1)+1} \qquad \text{S5.}$$

With the same condition and the largest root of λ_1, Eq. 11 becomes:

$$\begin{aligned} \frac{v}{x} &= \frac{1}{\lambda_1}\frac{\omega_1\lambda_1^3-(\omega_1-1)\lambda_1^2}{4\lambda_1^3-3(1+\omega_1 x)\lambda_1^2+2x(\omega_1-1)\lambda_1} = \frac{\omega_1\lambda_1-\omega_1+1}{4\lambda_1^2-3(1+\omega_1 x)\lambda_1+2x(\omega_1-1)} \\ &= \frac{\omega_1\lambda_1-\omega_1+1}{\lambda_1(4\lambda_1-3)+x(2\omega_1-2-3\omega_1\lambda_1)} \end{aligned} \qquad \text{S6.}$$

Substituting x from Eq. S5 into Eq. S6 yields:

$$(1-v)\omega_1\lambda_1^2-(-2\omega_1 v+2v+2\omega_1-1)\lambda_1+(1-v)(\omega_1-1)=0 \qquad \text{S7.}$$

As mentioned in (2), the physically meaningful root λ in Eq. S7 is

$$\begin{aligned} \lambda_+ &= \frac{-2\omega_1 v+2v+2\omega_1-1+\sqrt{(-2\omega_1 v+2v+2\omega_1-1)^2-4\omega_1(1-v)^2(\omega_1-1)}}{2\omega_1(1-v)} \\ &= \frac{(2\omega_1-1)(1-v)+v+R}{2\omega_1(1-v)} \\ &= \frac{(2\omega_1-1)^2(1-v)^2+2v(2\omega_1-1)(1-v)+v^2-R^2}{2\omega_1(1-v)\left((2\omega_1-1)(1-v)+v-R\right)} \\ &= \frac{2(\omega_1-1)(1-v)}{(2\omega_1-1)(1-v)+v-R} \end{aligned} \qquad \text{S8,}$$

where

$$
\begin{aligned}
R &= \sqrt{\left(-2\omega_1 v + 2v + 2\omega_1 - 1\right)^2 - 4\omega_1 \left(1-v\right)^2 \left(\omega_1 - 1\right)} \\
&= \sqrt{\left(2\omega_1 \left(1-v\right) + 2v - 1\right)^2 - 4\omega_1 \left(1-v\right)^2 \left(\omega_1 - 1\right)} \\
&= \sqrt{\left(1-2v\right)^2 + 4\omega_1 \left(1-v\right)\left(2v-1\right) + 4\omega_1^2 \left(1-v\right)^2 - 4\omega_1 \left(1-v\right)^2 \left(\omega_1 - 1\right)} \\
&= \sqrt{\left(1-2v\right)^2 + 4\omega_1 \left(1-v\right)\left(2v-1\right) + 4\omega_1 \left(1-v\right)^2} \\
&= \sqrt{\left(1-2v\right)^2 + 4\omega_1 \left(\left(2v-1\right) + \left(1-v\right)\right)\left(1-v\right)} \\
&= \sqrt{\left(1-2v\right)^2 + 4\omega_1 v \left(1-v\right)}
\end{aligned}
\qquad \text{S9.}
$$

Eq. S7 can be rewritten as

$$\omega_1 \lambda_1^2 + \left(1 - 2\omega_1 - \frac{v}{1-v}\right)\lambda_1 + \omega_1 - 1 = 0$$

i.e.

$$\frac{v}{1-v} = \frac{\lambda_1 - 1}{\lambda_1}\left(\omega_1 \lambda_1 - \omega_1 + 1\right) \qquad \text{S10}$$

Solving Eq. S5 for $\lambda_1 - 1$ with the largest root yields:

$$\lambda_1 - 1 = \frac{x\left(\omega_1\left(\lambda_1 - 1\right) + 1\right)}{\lambda_1} \qquad \text{S11}$$

Substituting Eq. S11 into Eq. S10, we find

$$\frac{v}{1-v} = x\left(\frac{\omega_1\left(\lambda_1 - 1\right) + 1}{\lambda_1}\right)^2 \qquad \text{S12}$$

Substituting Eq. S8 for λ_1 yields:

$$\frac{v}{1-v} = x\left(\frac{\omega_1 \dfrac{(2\omega_1-1)(1-v)+v+R}{2\omega_1(1-v)} - \omega_1 + 1}{\dfrac{(2\omega_1-1)(1-v)+v+R}{2\omega_1(1-v)}}\right)^2$$

$$= x\left(\frac{\omega_1(2\omega_1-1)(1-v)+v\omega_1+R\omega_1-2\omega_1^2(1-v)+2\omega_1(1-v)}{(2\omega_1-1)(1-v)+v+R}\right)^2$$

$$= x\left(\frac{2\omega_1^2(1-v)-2\omega_1^2(1-v)-\omega_1(1-v)+2\omega_1(1-v)+v\omega_1+R\omega_1}{(2\omega_1-1)(1-v)+v+R}\right)^2$$

$$= x\left(\omega_1\frac{R+1}{(2\omega_1-1)(1-v)+v+R}\right)^2$$

$$= x\left(\omega_1\frac{(R+1)\left((2\omega_1-1)(1-v)+v-R\right)}{\left((2\omega_1-1)(1-v)+vv\right)^2-R^2}\right)^2$$

$$= x\left(\omega_1\frac{(2\omega_1-1)(1-v)R+vR-R^2+(2\omega_1-1)(1-v)+v-R}{(2\omega_1-1)^2(1-v)^2+2v(2\omega_1-1)(1-v)+v^2-R^2}\right)^2$$

$$= x\left(\omega_1\frac{(2\omega_1-1)(1-v)R+vR-(1-2v)^2-4\omega_1 v(1-v)+(2\omega_1-1)(1-v)+v-R}{(2\omega_1-1)^2(1-v)^2+2v(2\omega_1-1)(1-v)+v^2-(1-2v)^2-4\omega_1 v(1-v)}\right)^2$$

$$= x\left(\omega_1\frac{(2\omega_1-1)(1-v)R+vR-R-(1-2v)^2-4\omega_1 v(1-v)+(2\omega_1-1)(1-v)+v}{(2\omega_1-1)^2(1-v)^2-2v(1-v)+v^2-(1-v)^2+2v(1-v)-v^2}\right)^2$$

$$= x\left(\omega_1\frac{(2\omega_1-1-1)(1-v)R-(1-2v)^2+2(1-2v)\omega_1(1-v)-(1-v)+v}{\left((2\omega_1-1)^2-1\right)(1-v)^2}\right)^2$$

$$= x\left(\omega_1\frac{2(\omega_1-1)(1-v)R+\left(-1+2v+2(1-v)\omega_1-1\right)(1-2v)}{4\omega_1(\omega_1-1)(1-v)^2}\right)^2$$

$$= x\left(\frac{2(\omega_1-1)(1-v)R+2(\omega_1-1)(1-v)(1-2v)}{4(\omega_1-1)(1-v)^2}\right)^2 = x\left(\frac{1-2v+R}{2(1-v)}\right)^2 \qquad \text{S13.}$$

Eq. S13 is identical to McGhee-von Hippel equation for ligand binding to a 1-D lattice with nearest neighbor interaction and a binding stoichiometry of 1 (2, 3). That is, when $\omega_2 = 1$, our analytic solution Eq. 11 reduces to McGhee-von Hippel equation for equilibrium ligand binding to 1-D lattice model.

3. *Solving characteristic equation Eq. 9 for x.*

For the largest root λ_1, characteristic equation (Eq. 9) is a quadratic equation for x according to the following:

$$\begin{aligned}
&\lambda_1^4 - (1+\omega_1\omega_2 x)\lambda_1^3 + \omega_2 x(\omega_1 - 1)\lambda_1^2 + x(\omega_2 - 1)(1+\omega_1\omega_2 x)\lambda_1 - x^2\omega_1(\omega_2-1)^2 \\
&= \lambda_1^4 - \lambda_1^3 + \left(-\omega_1\omega_2\lambda_1^2 + \omega_2(\omega_1-1)\lambda_1 + \omega_2 - 1\right)\lambda_1 x + (\omega_2-1)\left(\omega_1\omega_2\lambda_1 - \omega_1(\omega_2-1)\right)x^2 \\
&= \lambda_1^4 - \lambda_1^3 - \left((\omega_1\lambda_1+1)\omega_2(\lambda_1-1)+1\right)\lambda_1 x + \omega_1(\omega_2-1)\left(\omega_2(\lambda_1-1)+1\right)x^2 = 0
\end{aligned} \qquad \text{S14.}$$

The two roots of Eq. S14 are

$$\begin{aligned}
x &= \frac{\left((\omega_1\lambda_1+1)\omega_2(\lambda_1-1)+1\right)\lambda_1 \pm \sqrt{\left((\omega_1\lambda_1+1)\omega_2(\lambda_1-1)+1\right)^2\lambda_1^2 - 4\omega_1(\omega_2-1)\left(\omega_2(\lambda_1-1)+1\right)\lambda_1^3(\lambda_1-1)}}{2\omega_1(\omega_2-1)\left(\omega_2(\lambda_1-1)+1\right)} \\
&= \lambda_1\frac{(\omega_1\lambda_1+1)\omega_2(\lambda_1-1)+1 \pm \sqrt{\left((\omega_1\lambda_1+1)\omega_2(\lambda_1-1)+1\right)^2 - 4\omega_1(\omega_2-1)\left(\omega_2(\lambda_1-1)+1\right)\lambda_1(\lambda_1-1)}}{2\omega_1(\omega_2-1)\left(\omega_2(\lambda_1-1)+1\right)}
\end{aligned} \qquad \text{S15.}$$

When $\omega_2 \to 1$, one of the two roots, x_+ approaches infinity:

$$\begin{aligned}
x_+ &= \lambda_1\frac{(\omega_1\lambda_1+1)\omega_2(\lambda_1-1)+1 + \sqrt{\left((\omega_1\lambda_1+1)\omega_2(\lambda_1-1)+1\right)^2 - 4\omega_1(\omega_2-1)\left(\omega_2(\lambda_1-1)+1\right)\lambda_1(\lambda_1-1)}}{2\omega_1(\omega_2-1)\left(\omega_2(\lambda_1-1)+1\right)} \\
&\xrightarrow{\omega_2\to 1} \lambda_1\frac{(\omega_1\lambda_1+1)\omega_2(\lambda_1-1)+1}{\omega_1(\omega_2-1)\left(\omega_2(\lambda_1-1)+1\right)} \to \infty
\end{aligned} \qquad \text{S16,}$$

and therefore, x_+ is not a physically meaningful root and the only physically meaningful root, x_- is given by:

$$x_{-}=\lambda_1\frac{(\omega_1\lambda_1+1)\omega_2(\lambda_1-1)+1-\sqrt{((\omega_1\lambda_1+1)\omega_2(\lambda_1-1)+1)^2-4\omega_1(\omega_2-1)(\omega_2(\lambda_1-1)+1)\lambda_1(\lambda_1-1)}}{2\omega_1(\omega_2-1)(\omega_2(\lambda_1-1)+1)}$$

$$=\frac{2\lambda_1^2(\lambda_1-1)}{(\omega_1\lambda_1+1)\omega_2(\lambda_1-1)+1+\sqrt{((\omega_1\lambda_1+1)\omega_2(\lambda_1-1)+1)^2-4\omega_1(\omega_2-1)(\omega_2(\lambda_1-1)+1)\lambda_1(\lambda_1-1)}} \qquad \text{S17}$$

and

$$\xrightarrow{\omega_2\to 1}\lambda_1\frac{\lambda_1(\lambda_1-1)}{(\omega_1\lambda_1+1)(\lambda_1-1)+1}=\lambda_1\frac{\lambda_1(\lambda_1-1)}{\omega_1\lambda_1^2+\lambda_1-\omega_1\lambda_1}=\lambda_1\frac{\lambda_1-1}{\omega_1(\lambda_1-1)+1} \qquad \text{S18}$$

Note that Eq. S18 is identical to Eq. S5.

From Eq. S17, an upper limit of λ at a given total ligand concentration L_t can be estimated:

$$x=\frac{L_f}{K_d}=\frac{2\lambda_1^2(\lambda_1-1)}{(\omega_1\lambda_1+1)\omega_2(\lambda_1-1)+1+\sqrt{((\omega_1\lambda_1+1)\omega_2(\lambda_1-1)+1)^2-4\omega_1(\omega_2-1)(\omega_2(\lambda_1-1)+1)\lambda_1(\lambda_1-1)}}<\frac{L_t}{K_d}$$

i.e.,

$$\begin{aligned}\lambda_1^3&<\lambda_1^2+\frac{L_t}{2K_d}\left((\omega_1\lambda_1+1)\omega_2(\lambda_1-1)+1+\sqrt{((\omega_1\lambda_1+1)\omega_2(\lambda_1-1)+1)^2-4\omega_1(\omega_2-1)(\omega_2(\lambda_1-1)+1)\lambda_1(\lambda_1-1)}\right)\\&<\lambda_1^2+\frac{L_t}{2K_d}\left((\omega_1\lambda_1+1)\omega_2(\lambda_1-1)+1+\sqrt{((\omega_1\lambda_1+1)\omega_2(\lambda_1-1)+1)^2}\right)\\&<\lambda_1^2+\frac{L_t}{K_d}((\omega_1\lambda_1+1)\omega_2(\lambda_1-1)+1)=\lambda_1^2+\frac{L_t}{K_d}\left(\omega_1\omega_2\lambda_1^2+\omega_2\lambda_1-\omega_1\omega_2\lambda_1-\omega_2+1\right)\\&<\lambda_1^2+\frac{L_t}{K_d}\left(\omega_1\omega_2\lambda_1^2+\omega_2\lambda_1+1\right)\ .\end{aligned}$$

Since $\lambda_1 \geq 1$, it can be expressed as:

$$\lambda_1<1+\frac{L_t}{K_d}(\omega_1\omega_2+\omega_2+1) \qquad \text{S19}$$

This estimated λ_1 upper limit value, together with λ_1 lower limit value of 1, is very useful to bracket the root in numerically solving Eq. 9 for the largest root.

4. *Several derivatives used in numerical procedures to solve Eq. 25 for simulation and data analysis.*

The physically meaningful value of x for a given $\lambda \geq 1$ is given by Eq. S17. The derivative of Eq. S17 leads to

$$
\begin{aligned}
\frac{dx}{d\lambda} &= \frac{2\lambda_1(3\lambda_1-2)}{(\omega_1\lambda_1+1)\omega_2(\lambda_1-1)+1+\sqrt{((\omega_1\lambda_1+1)\omega_2(\lambda_1-1)+1)^2-4\omega_1(\omega_2-1)(\omega_2(\lambda_1-1)+1)\lambda_1(\lambda_1-1)}} \\
&\quad - \frac{2\lambda_1^{2}(\lambda_1-1)\left(\omega_2(\omega_1(2\lambda_1-1)+1)+\frac{2((\omega_1\lambda_1+1)\omega_2(\lambda_1-1)+1)(\omega_1\omega_2(\lambda_1-1)+(\omega_1\lambda_1+1)\omega_2)-4\omega_1\omega_2(\omega_2-1)\lambda_1(\lambda_1-1)-4\omega_1(\omega_2-1)(\omega_2(\lambda_1-1)+1)(2\lambda_1-1)}{2\sqrt{((\omega_1\lambda_1+1)\omega_2(\lambda_1-1)+1)^2-4\omega_1(\omega_2-1)(\omega_2(\lambda_1-1)+1)\lambda_1(\lambda_1-1)}}\right)}{\left((\omega_1\lambda_1+1)\omega_2(\lambda_1-1)+1+\sqrt{((\omega_1\lambda_1+1)\omega_2(\lambda_1-1)+1)^2-4\omega_1(\omega_2-1)(\omega_2(\lambda_1-1)+1)\lambda_1(\lambda_1-1)}\right)^2} \\
&= \frac{2\lambda_1(3\lambda_1-2)}{(\omega_1\lambda_1+1)\omega_2(\lambda_1-1)+1+\sqrt{((\omega_1\lambda_1+1)\omega_2(\lambda_1-1)+1)^2-4\omega_1(\omega_2-1)(\omega_2(\lambda_1-1)+1)\lambda_1(\lambda_1-1)}} \\
&\quad - \frac{2\lambda_1^{2}(\lambda_1-1)\left(\omega_2(\omega_1(2\lambda_1-1)+1)+\frac{\omega_2(\omega_2(\omega_1\lambda_1+1)(\lambda_1-1)+1)(\omega_1(2\lambda_1-1)+1)-2\omega_1(\omega_2-1)(\omega_2\lambda_1(\lambda_1-1)+(\omega_2(\lambda_1-1)+1)(2\lambda_1-1))}{\sqrt{((\omega_1\lambda_1+1)\omega_2(\lambda_1-1)+1)^2-4\omega_1(\omega_2-1)(\omega_2(\lambda_1-1)+1)\lambda_1(\lambda_1-1)}}\right)}{\left((\omega_1\lambda_1+1)\omega_2(\lambda_1-1)+1+\sqrt{((\omega_1\lambda_1+1)\omega_2(\lambda_1-1)+1)^2-4\omega_1(\omega_2-1)(\omega_2(\lambda_1-1)+1)\lambda_1(\lambda_1-1)}\right)^2}
\end{aligned}
$$

S20.

Taking derivatives of v to x and v to λ at both sides of Eq. 11 in the main text yields:

$$
\begin{aligned}
\frac{\partial v}{\partial x} &= \frac{1}{\lambda_1}\frac{\omega_1\omega_2\lambda_1^3-\omega_2(\omega_1-1)\lambda_1^2-(\omega_2-1)(1+2\omega_1\omega_2 x)\lambda_1+2x\omega_1(\omega_2-1)^2}{4\lambda_1^3-3(1+\omega_1\omega_2 x)\lambda_1^2+2\omega_2 x(\omega_1-1)\lambda_1+x(\omega_2-1)(1+\omega_1\omega_2 x)} \\
&\quad + \frac{x}{\lambda_1}\frac{2\omega_1(\omega_2-1)(\omega_2(1-\lambda_1)-1)}{4\lambda_1^3-3(1+\omega_1\omega_2 x)\lambda_1^2+2\omega_2 x(\omega_1-1)\lambda_1+x(\omega_2-1)(1+\omega_1\omega_2 x)}
\end{aligned}
$$

$$-\frac{x}{\lambda_1}\frac{\left(\omega_1\omega_2\lambda_1^3-\omega_2(\omega_1-1)\lambda_1^2-(\omega_2-1)(1+2\omega_1\omega_2x)\lambda_1+2x\omega_1(\omega_2-1)^2\right)\times\left(-3\omega_1\omega_2\lambda_1^2+2\omega_2(\omega_1-1)\lambda_1+(\omega_2-1)(1+2\omega_1\omega_2x)\right)}{\left(4\lambda_1^3-3(1+\omega_1\omega_2x)\lambda_1^2+2\omega_2x(\omega_1-1)\lambda_1+x(\omega_2-1)(1+\omega_1\omega_2x)\right)^2} \quad \text{S21}$$

$$\begin{aligned}\frac{\partial v}{\partial\lambda_1}=&-\frac{x}{\lambda_1^2}\frac{\omega_1\omega_2\lambda_1^3-\omega_2(\omega_1-1)\lambda_1^2-(\omega_2-1)(1+2\omega_1\omega_2x)\lambda_1+2x\omega_1(\omega_2-1)^2}{4\lambda_1^3-3(1+\omega_1\omega_2x)\lambda_1^2+2\omega_2x(\omega_1-1)\lambda_1+x(\omega_2-1)(1+\omega_1\omega_2x)}\\&+\frac{x}{\lambda_1}\frac{3\omega_1\omega_2\lambda_1^2-2\omega_2(\omega_1-1)\lambda_1-(\omega_2-1)(1+2\omega_1\omega_2x)}{4\lambda_1^3-3(1+\omega_1\omega_2x)\lambda_1^2+2\omega_2x(\omega_1-1)\lambda_1+x(\omega_2-1)(1+\omega_1\omega_2x)}\\&-\frac{x}{\lambda_1}\frac{2\left(\omega_1\omega_2\lambda_1^3-\omega_2(\omega_1-1)\lambda_1^2-(\omega_2-1)(1+2\omega_1\omega_2x)\lambda_1+2x\omega_1(\omega_2-1)^2\right)\times\left(6\lambda_1^2-3(1+\omega_1\omega_2x)\lambda_1+\omega_2x(\omega_1-1)\right)}{\left(4\lambda_1^3-3(1+\omega_1\omega_2x)\lambda_1^2+2\omega_2x(\omega_1-1)\lambda_1+x(\omega_2-1)(1+\omega_1\omega_2x)\right)^2}\end{aligned} \quad \text{S22.}$$

5. *Bound ligand clusters*

When $x = L_t = L = 0$, the largest root is $\lambda_1 = 1$ (Eq. S2) and the average bound ligand cluster size with and without single empty site gaps is given by:

$$\begin{aligned}\overline{C}_{sgap}&=\frac{\omega_1\omega_2\lambda_1^3+\omega_2(2-\omega_1)\lambda_1^2-\left((\omega_2-1)(1+3\omega_1\omega_2x)+\omega_2\right)\lambda_1+(3\omega_2-2)\omega_1x(\omega_2-1)}{\lambda_1-\omega_1x(\omega_2-1)}\\&\xrightarrow{x=0,\lambda_1=1}\frac{\omega_1\omega_2+\omega_2(2-\omega_1)-\left((\omega_2-1)+\omega_2\right)}{1}=1\end{aligned} \quad \text{S23}$$

$$\begin{aligned}\overline{C}_{nogap}&=\frac{\omega_1\omega_2\lambda_1^3-\omega_2(\omega_1-1)\lambda_1^2-(\omega_2-1)(1+2\omega_1\omega_2x)\lambda_1+2x\omega_1(\omega_2-1)^2}{\omega_2\lambda^2-(\omega_2\omega_1x+1)(\omega_2-1)\lambda_1+\omega_1x(\omega_2-1)^2}\\&\to\frac{\omega_1\omega_2-\omega_2(\omega_1-1)-(\omega_2-1)}{\omega_2-(\omega_2-1)}=1\end{aligned} \quad \text{S24}$$

When $\omega_2 = 1$, the average bound ligand cluster size with and without single empty site gaps is given by:

$$\overline{C}_{sgap} = \frac{\omega_1 \lambda_1^{\,3} + (2-\omega_1)\lambda_1^{\,2} - (0+1)\lambda_1 + 0}{\lambda_1 - 0} = \omega_1 \lambda_1^{\,2} + (2-\omega_1)\lambda_1 - 1$$

$$= \omega_1 \left(\frac{2(\omega_1 - 1)(1-v)}{(2\omega_1 - 1)(1-v) + v - R} \right)^2 + (2-\omega_1) \frac{2(\omega_1 - 1)(1-v)}{(2\omega_1 - 1)(1-v) + v - R} - 1$$

$$= \omega_1 \frac{2(\omega_1 - 1)(1-v)}{(2\omega_1 - 1)(1-v) + v - R} \frac{(2\omega_1 - 1)(1-v) + v + R}{2\omega_1 (1-v)} + (2-\omega_1) \frac{2(\omega_1 - 1)(1-v)}{(2\omega_1 - 1)(1-v) + v - R} - 1$$

$$= \frac{(\omega_1 - 1)\big((2\omega_1 - 1)(1-v) + v + R\big) + 2(2-\omega_1)(\omega_1 - 1)(1-v) - (2\omega_1 - 1)(1-v) - v + R}{(2\omega_1 - 1)(1-v) + v - R}$$

$$= \frac{\omega_1 (1+R) - 2}{(2\omega_1 - 1)(1-v) + v - R} = \begin{cases} \dfrac{\omega_1 (1+1) - 2}{(2\omega_1 - 1)(1-0) + 0 - 1} = 1, & v = 0 \\ \dfrac{\omega_1 (1+1) - 2}{(2\omega_1 - 1)(1-1) + 1 - 1} \to \infty, & v \to 1 \end{cases} \qquad \text{S25}$$

$$\overline{C}_{nogap} = \frac{\omega_1 \lambda_1^{\,3} - (\omega_1 - 1)\lambda_1^{\,2} - 0 + 0}{\lambda^2 - 0 + 0} = \omega_1 \lambda_1 - (\omega_1 - 1) = \omega_1 (\lambda_1 - 1) + 1$$

$$= \omega_1 \left(\frac{(2\omega_1 - 1)(1-v) + v + R}{2\omega_1 (1-v)} - 1 \right) + 1$$

$$= \omega_1 \left(\frac{(2\omega_1 - 1)(1-v) + v + R - 2\omega_1 (1-v)}{2\omega_1 (1-v)} \right) + 1$$

$$= \frac{-1 + 2v + R}{2(1-v)} + 1 = \frac{-1 + 2v + R + 2(1-v)}{2(1-v)} = \frac{1+R}{2(1-v)} = \begin{cases} \dfrac{1+1}{2(1-0)} = 1, & v = 0 \\ \dfrac{1+1}{2(1-1)} \to \infty, & v \to 1 \end{cases}$$

$$= \frac{R^2 - 1}{2(1-v)(R-1)} = \frac{(1-2v)^2 + 4\omega_1 v(1-v) - 1}{2(1-v)(R-1)}$$

$$= \frac{1 - 4v + 4v^2 + 4\omega_1 v(1-v) - 1}{2(1-v)(R-1)} = \frac{-4v(1-v) + 4\omega_1 v(1-v)}{2(1-v)(R-1)}$$

$$= \frac{4v(\omega_1 - 1)(1-v)}{2(1-v)(R-1)} = \frac{2v(\omega_1 - 1)}{R-1} \qquad \text{S26}$$

λ_1 from Eq. S8 is substituted into Eqs. S25 and S26.

The last expression in Eq. S26 is exactly identical to the result previously derived using the conditional probability method in 1-D lattice model (4). However, this expression has singularity at both $\nu = 0$ and 1 since $R = 1$ at those 2 ν points, and the singularity at $\nu = 0$ would not be there if using the middle expression where the average cluster size values for binding density $\nu = 0$ and 1 are. Below (Eq. S34) will show that Eq. S25 is exactly identical to that derived using the conditional probability method in 1-D lattice model (3) as well.

6. *Data lysis and average bound ligand cluster size by 1-D Ising model*

The binding equation previously derived by the conditional probability method (3) for ligand cooperative binding to 1-D Ising lattice was used under condition of stoichiometric binding (n =1) for data analysis and cluster size calculation. To fit experimental equilibrium ligand binding titration curves plotted as signal ($\propto \nu$) vs. L_t, the value of ν at a given L_t value was numerically solved with equation (3) combined with ligand mass conservation:

$$\frac{\nu}{L_t - \nu A_t} = \frac{(1-n\nu)}{K_d}\left(\frac{(2\omega-1)(1-n\nu)+\nu-R}{2(\omega-1)(1-n\nu)}\right)^{n-1}\left(\frac{1-(n+1)\nu+R}{2(1-n\nu)}\right)^2, \quad \omega \neq 1 \qquad 27$$

This equation has a singularity at $\omega = 1$, and this singularity can be removed by re-writing the equation as following:

$$\frac{\nu}{L_t - \nu A_t} = \frac{(1-n\nu)}{K_d}\left(\frac{2\omega(1-n\nu)}{(2\omega-1)(1-n\nu)+\nu+R}\right)^{n-1}\left(\frac{1-(n+1)\nu+R}{2(1-n\nu)}\right)^2, \quad 0<\omega<\infty \qquad 28$$

In this form, $\omega = 1$ is no longer a singularity. In the two equations above

$$R = \sqrt{(1-n\nu-\nu)^2 + 4\omega\nu(1-n\nu)} \qquad 29.$$

When $n = 1$, Eq. 27 or 28 can be analytically solved for ν as function of the *free* ligand concentration L_f (see Eq. S58 below). However, Eq. 27 or 28 cannot be solved analytically for ν as function of the *total* ligand L_t because L_f is unknown as well. In this case, a numerical procedure has to be employed.

The average cluster size in the 1-D Ising model without any gaps has been previously derived (4) and the expression is given by Eqs. 21 and S26. The average cluster size in the 1-D Ising

model with single empty site gaps was not previously derived, but is presented here (Eq. S25) as a special case (ω_2 = 1) in the 2-D Ising model. It can also be directly derived with the conditional probability method used in the 1-D model (presented below).

From the conditional probability method (3), the probability of one empty site gap (g = 1 gap) next to a bound ligand is defined by:

$$P_{g=1} = (b_n f)(fb_1) \qquad \text{S30,}$$

and the average number of singly isolated unoccupied sites is given by:

$$\bar{N}_{bound} P_{g=1} = \bar{N}_{bound}(b_n f)(fb_1),$$

where $\bar{N}_{bound} = N\nu$ is average number of bound ligands. Thus, the number of bound ligands plus singly isolated unoccupied gaps is given by:

$$\bar{N}_{bound} + \bar{N}_{bound}(b_n f)(fb_1) = N\nu\left(1+(b_n f)(fb_1)\right) \qquad \text{S31.}$$

The probability of finding any empty site gap ≥2 next to a bound ligand is given by the sum of all of probabilities of empty site gaps with length ≥2:

$$\sum_{g=2}^{\infty} P_g = 1 - P_0 - P_1 = 1-(b_n b_1)-(b_n f)(fb_1) = (b_n f)-(b_n f)(fb_1) = (b_n f)\left(1-(fb_1)\right) = (b_n f)(ff) \qquad \text{S32}$$

where P_1 is given by S30 and $P_0 = (b_n b_1)$ is the probability of not finding any empty site gap (or gap = 0) next to a bound ligand. The number of all gaps ≥ 2 is thus given by:

$$\bar{N}_{bound} \sum_{g=2}^{\infty} P_g = N\nu(b_n f)(ff) \qquad \text{S33,}$$

which is equal to the number of bound ligand clusters with the single gaps in an infinite lattice. Therefore, according to Eqs. S31 and S33, the average length of clusters with singly isolated unoccupied site gaps ($\bar{C}_{sgap}$) is expressed as:

$$
\begin{aligned}
\bar{C}_{sgap} &= \frac{\text{bound ligands+single gaps}}{\text{number of clusters of bound ligand with single gap}} = \frac{N\nu\left(1+\left(b_n f\right)\left(fb_1\right)\right)}{N\nu\left(b_n f\right)\left(ff\right)} \\
&= \frac{1+\dfrac{(n-1)\nu-1+R}{2\nu(\omega-1)}\dfrac{(n-1)\nu-1+R}{2(\omega-1)(1-n\nu)}}{\dfrac{(n-1)\nu-1+R}{2\nu(\omega-1)}\dfrac{(2\omega-1)(1-n\nu)+\nu-R}{2(\omega-1)(1-n\nu)}}, \quad n=1 \\
&= \frac{1+\dfrac{-1+R}{2\nu(\omega-1)}\dfrac{-1+R}{2(\omega-1)(1-\nu)}}{\dfrac{-1+R}{2\nu(\omega-1)}\dfrac{(2\omega-1)(1-\nu)+\nu-R}{2(\omega-1)(1-\nu)}} \\
&= \frac{\omega(R+1)-2}{(2\omega-1)(1-\nu)+\nu-R} \qquad \text{S34,}
\end{aligned}
$$

where the term R is the same as that in Eqs. S9 and 13 (in main text).

Another way to calculate the average number of bound ligand clusters with single site gaps is to calculate the average number of bound ligands with at least 2 empty sites at their *right*, $\bar{N}_{bound}\left(b_n f\right)\left(ff\right)$, similar to the method used previously (4). We can count the average number of bound ligands with at least 2 empty sites at their *left* as well, that is, $\left(N-\bar{N}_{bound}\right)\left(ff\right)\left(fb_1\right)=N\left(1-\nu\right)\left(ff\right)\left(fb_1\right)$, where $N-\bar{N}_{bound}=N\left(1-\nu\right)$ is the total number of empty sites. The last expression is identical to the previous one and to Eq. S33.

7. *Solving ligand binding to the double stranded lattice model by the transfer matrix method*

We use the linearized double-stranded Ising model (Fig. 1C) to build the grand partition function. In the transfer matrix approach (2, 5-7), it is convenient to model the infinite long polymer as a circular Ising lattice (2). When the number of lattice binding sites $N \to \infty$, the grand partition functions of circular and non-circular lattice are identical. Therefore, we use a circular Ising lattice (i.e. the first site (i = 1) is adjacent to the last one (i = N)). The grand partition function for a circular lattice of N ligand binding sites is given by:

$$\Xi = \sum_{s_1=0}^{1} \cdots \sum_{s_N=0}^{1} x^{s_1+\ldots+s_N} \omega_1^{s_1 s_2} \ldots \omega_1^{s_{N-1} s_N} \omega_1^{s_N s_1} \omega_2^{s_1 s_3} \omega_2^{s_2 s_4} \ldots \omega_2^{s_{N-2} s_N} \omega_2^{s_{N-1} s_1} \omega_2^{s_N s_2}$$

$$= \begin{cases} \sum_{s_1=0}^{1} \cdots \sum_{s_N=0}^{1} \left(x^{s_1+s_2} \omega_1^{s_1 s_2} \omega_1^{s_2 s_3} \omega_2^{s_1 s_3} \omega_2^{s_2 s_4} \right) \left(x^{s_3+s_4} \omega_1^{s_3 s_4} \omega_1^{s_4 s_5} \omega_2^{s_3 s_5} \omega_2^{s_4 s_6} \right) \ldots \\ \qquad \left(x^{s_{N-1}+s_N} \omega_1^{s_{N-1} s_N} \omega_1^{s_N s_1} \omega_2^{s_{N-1} s_1} \omega_2^{s_N s_2} \right), \qquad \text{when } N \text{ is even} \\ \sum_{s_1=0}^{1} \cdots \sum_{s_N=0}^{1} \left(x^{s_1+s_2} \omega_1^{s_1 s_2} \omega_1^{s_2 s_3} \omega_2^{s_1 s_3} \omega_2^{s_2 s_4} \right) \left(x^{s_3+s_4} \omega_1^{s_3 s_4} \omega_1^{s_4 s_5} \omega_2^{s_3 s_5} \omega_2^{s_4 s_6} \right) \ldots \\ \left(x^{s_{N-2}+s_{N-1}} \omega_1^{s_{N-2} s_{N-1}} \omega_1^{s_{N-1} s_N} \omega_2^{s_{N-2} s_N} \omega_2^{s_{N-1} s_1} \right) \left(x^{s_N} \omega_1^{s_N s_1} \omega_2^{s_N s_2} \right), \qquad \text{when } N \text{ is odd} \end{cases}$$

S35

where $s_i = \{0,1\}$, $i = 1, 2, \ldots N$, for binding site i to be empty or occupied by a bound ligand; $x = K_a L_f = \frac{L_f}{K_d}$ in which K_a and K_d is intrinsic equilibrium association and dissociation binding constant for ligand binding to an individual site, and L_f is free ligand concentration; ω_1 and ω_2 are the cooperativity parameters for binding to the nearest and the second nearest neighbor sites from a bound ligand, respectively. The last expression of the grand partition function (Eq. S35), has been grouped by every 4 lattice binding sites (e.g. site 1-4) for later use (see below).

The smallest individual interaction unit in the first equation of Eq. S35 is a single lattice binding site, which can exist in two states: with and without a bound ligand. A ligand-occupied site can interact with its nearest and second nearest neighbors. We redefine the smallest interaction unit in the second equation of Eq. S35 as two consecutive lattice binding sites (i.e., make every two adjacent binding sits as an inseparable unit and the total number of the interaction units now (m) becomes a half of the original number of the units (N), i.e., $m = N/2$ when N is even or N–1 when N is odd). This new unit can exist in 4 discrete states, which reflect combination of states of the two composed sites (i.e., $\{s_1, s_2\} = \{(0, 0), (1, 0), (0, 1), (1, 1)\}$ (Table S1)).

We define a 4×4 transfer matrix (P) with its components as follows, which is a grouped 4 lattice sites bracketed with a pair of parentheses in Eq. S35:

$$P_{v_k v_{k+1}} = x^{s_{2k-1}+s_{2k}} \omega_1^{s_{2k-1} s_{2k}} \omega_1^{s_{2k} s_{2k+1}} \omega_2^{s_{2k-1} s_{2k+1}} \omega_2^{s_{2k} s_{2k+2}}$$
$$= \exp\left(\left(s_{2k-1} + s_{2k} \right) \ln x + \left(s_{2k-1} s_{2k} + s_{2k} s_{2k+1} \right) \ln \omega_1 + \left(s_{2k-1} s_{2k+1} + s_{2k} s_{2k+2} \right) \ln \omega_2 \right) \qquad \text{S36.}$$

The matrix indices v_k, v_{k+1} = 1, 2, 3, 4. In Eq. S36, k = 1, 2, …, $m = N/2$ (N is even) or (N–1) (N is odd) is an index marking k th unit from the m number of units, and every unit has 4 states indicated by the matrix index v_k = 1, …, 4. For the states at kth unit, the 4 states are listed in Table S1:

Table S1. States of *k*th new unit	
v_k	$\{s_1, s_2\}$
1	0, 0
2	1, 0
3	0, 1
4	1, 1

Accordingly, the transfer matrix is given by:

$$P = \begin{pmatrix} 1 & 1 & 1 & 1 \\ x & x\omega_2 & x & x\omega_2 \\ x & x\omega_1 & x\omega_2 & x\omega_1\omega_2 \\ x^2\omega_1 & x^2\omega_1{}^2\omega_2 & x^2\omega_1\omega_2 & x^2\omega_1{}^2\omega_2{}^2 \end{pmatrix} \qquad \text{S37}$$

The transfer matrix P has 4 eigenvalues, denoted as λ_i, i = 1, …, 4, which we arrange by descending absolute values, i.e., $|\lambda_1| > |\lambda_2| > \ldots > |\lambda_4|$. We let matrix T be a matrix with its column vectors given by eigenvectors of transfer matrix P, and T^{-1} be T's inverse matrix, such that, P is defined by the transfer matrix and its eigenvalues by the following:

$$PT = T\begin{pmatrix} \lambda_1 & & & \\ & \lambda_2 & & \\ & & \lambda_3 & \\ & & & \lambda_4 \end{pmatrix} \text{ or } P = T\begin{pmatrix} \lambda_1 & & & \\ & \lambda_2 & & \\ & & \lambda_3 & \\ & & & \lambda_4 \end{pmatrix}T^{-1} \qquad \text{S38.}$$

Using the transfer matrix P (Eq. S37), invertible matrix T, and eigenvalues λ_i, the grand partition function Eq. S35 becomes Eq. 39 when N is even and $m = N/2$ or Eq. 40 when N is odd and m = (N–1)/2, as follows:

$$\Xi = \sum_{v_1=1}^{4}\cdots\sum_{v_m=1}^{4} P_{v_1v_2}P_{v_2v_3}\ldots P_{v_mv_1} = \mathrm{tr}\left(P^m\right)$$

$$= \mathrm{tr}\left(\left(T\begin{pmatrix}\lambda_1 & & & \\ & \lambda_2 & & \\ & & \lambda_3 & \\ & & & \lambda_4\end{pmatrix}T^{-1}\right)^m\right) = \mathrm{tr}\left(T\begin{pmatrix}\lambda_1{}^m & & & \\ & \lambda_2{}^m & & \\ & & \lambda_3{}^m & \\ & & & \lambda_4{}^m\end{pmatrix}T^{-1}\right)$$

$$= \mathrm{tr}\left(\begin{pmatrix}\lambda_1{}^m & & & \\ & \lambda_2{}^m & & \\ & & \lambda_3{}^m & \\ & & & \lambda_4{}^m\end{pmatrix}\right) = \lambda_1{}^m + \lambda_2{}^m + \lambda_3{}^m + \lambda_4{}^m \qquad \text{S39}$$

$$\Xi = \sum_{v_1=1}^{4}\cdots\sum_{v_m=1}^{4}\sum_{s_N=0}^{1} P_{v_1v_2}P_{v_2v_3}\ldots P_{v_{m-1}v_m}\left(x^{s_{N-2}+s_{N-1}}\omega_1{}^{s_{N-2}s_{N-1}}\omega_1{}^{s_{N-1}s_N}\omega_2{}^{s_{N-2}s_N}\omega_2{}^{s_{N-1}s_1}\right)\left(x^{s_N}\omega_1{}^{s_Ns_1}\omega_2{}^{s_Ns_2}\right)$$

$$= \sum_{v_1=1}^{4}\cdots\sum_{v_m=1}^{4}\sum_{s_N=0}^{1} P_{v_1v_2}P_{v_2v_3}\ldots P_{v_{m-1}v_m}P_{v_m\{s_N,s_1\}}\left(x^{s_N}\omega_1{}^{s_Ns_1}\omega_2{}^{s_Ns_2}\right)$$

$$= \sum_{v_1=1}^{4}\sum_{s_N=0}^{1}\left(P^m\right)_{v_1\{s_N,s_1\}}\left(x^{s_N}\omega_1{}^{s_Ns_1}\omega_2{}^{s_Ns_2}\right)$$

$$= P^m{}_{11} + xP^m{}_{12} + P^m{}_{23} + x\omega_1P^m{}_{24} + P^m{}_{31} + x\omega_2P^m{}_{32} + P^m{}_{43} + x\omega_1\omega_2P^m{}_{44}$$

$$= \mathrm{tr}\left(P^m\begin{pmatrix}1 & 0 & 1 & 0\\ x & 0 & x\omega_2 & 0\\ 0 & 1 & 0 & 1\\ 0 & x\omega_1 & 0 & x\omega_1\omega_2\end{pmatrix}\right) = \mathrm{tr}\left(\left(T\begin{pmatrix}\lambda_1 & & & \\ & \lambda_2 & & \\ & & \lambda_3 & \\ & & & \lambda_4\end{pmatrix}T^{-1}\right)^m\begin{pmatrix}1 & 0 & 1 & 0\\ x & 0 & x\omega_2 & 0\\ 0 & 1 & 0 & 1\\ 0 & x\omega_1 & 0 & x\omega_1\omega_2\end{pmatrix}\right)$$

$$=\mathrm{tr}\left(T\begin{pmatrix}\lambda_1{}^m & & & \\ & \lambda_2{}^m & & \\ & & \lambda_3{}^m & \\ & & & \lambda_4{}^m\end{pmatrix}T^{-1}\begin{pmatrix}1 & 0 & 1 & 0\\ x & 0 & x\omega_2 & 0\\ 0 & 1 & 0 & 1\\ 0 & x\omega_1 & 0 & x\omega_1\omega_2\end{pmatrix}\right)$$

$$= \mathrm{tr}\left(\begin{pmatrix}\lambda_1{}^m & & & \\ & \lambda_2{}^m & & \\ & & \lambda_3{}^m & \\ & & & \lambda_4{}^m\end{pmatrix}T^{-1}\begin{pmatrix}1 & 0 & 1 & 0\\ x & 0 & x\omega_2 & 0\\ 0 & 1 & 0 & 1\\ 0 & x\omega_1 & 0 & x\omega_1\omega_2\end{pmatrix}T\right) = \mathrm{tr}\left(\begin{pmatrix}\lambda_1{}^m & & & \\ & \lambda_2{}^m & & \\ & & \lambda_3{}^m & \\ & & & \lambda_4{}^m\end{pmatrix}C\right)$$

$$= \lambda_1{}^m C_{11} + \lambda_2{}^m C_{22} + \lambda_3{}^m C_{33} + \lambda_4{}^m C_{44} \qquad \text{S40}$$

Matrix C is x-, ω_1- and ω_2-dependent and defined as:

$$C = T^{-1} \begin{pmatrix} 1 & 0 & 1 & 0 \\ x & 0 & x\omega_2 & 0 \\ 0 & 1 & 0 & 1 \\ 0 & x\omega_1 & 0 & x\omega_1\omega_2 \end{pmatrix} T \qquad \text{S41}$$

If $\bar{N}_{bound}$ is average of number of sites bound with ligand, the average number of bound ligand per site/ligand occupancy/binding density is given by:

$$\nu = \frac{\bar{N}_{bound}}{N}$$
$$= \frac{1}{N\Xi} \sum_{s_1=0}^{1} \cdots \sum_{s_N=0}^{1} \left(s_1 + \ldots + s_N \right) x^{s_1 + \ldots + s_N} \omega_1^{s_1 s_2} \ldots \omega_2^{s_{N-1} s_N} \omega_2^{s_N s_1} \omega_2^{s_1 s_3} \omega_2^{s_2 s_4} \ldots \omega_2^{s_{N-2} s_N} \omega_2^{s_{N-1} s_1} \omega_2^{s_N s_2}$$
$$= \frac{x}{N} \frac{\partial \ln \Xi}{\partial x} \qquad \text{S42}$$

When N is even and $m = N/2$, the grand partition function is expressed in Eq. S39 and the binding density in Eq. S42 becomes:

$$\nu = \frac{x}{N} \frac{\partial}{\partial x} \ln \left(\lambda_1^{\frac{N}{2}} + \lambda_2^{\frac{N}{2}} + \lambda_3^{\frac{N}{2}} + \lambda_4^{\frac{N}{2}} \right)$$
$$= \frac{x}{N} \frac{\left(\frac{N}{2} \lambda_1^{\frac{N}{2}-1} \frac{\partial \lambda_1}{\partial x} + \frac{N}{2} \lambda_2^{\frac{N}{2}-1} \frac{\partial \lambda_2}{\partial x} + \frac{N}{2} \lambda_3^{\frac{N}{2}-1} \frac{\partial \lambda_3}{\partial x} + \frac{N}{2} \lambda_4^{\frac{N}{2}-1} \frac{\partial \lambda_4}{\partial x} \right)}{\lambda_1^{\frac{N}{2}} + \lambda_2^{\frac{N}{2}} + \lambda_3^{\frac{N}{2}} + \lambda_4^{\frac{N}{2}}}$$

$$= \frac{x}{2\lambda_1}\frac{\left(\frac{\partial \lambda_1}{\partial x} + \left(\frac{\lambda_2}{\lambda_1}\right)^{\frac{N}{2}-1}\frac{\partial \lambda_2}{\partial x} + \left(\frac{\lambda_3}{\lambda_1}\right)^{\frac{N}{2}-1}\frac{\partial \lambda_3}{\partial x} + \left(\frac{\lambda_4}{\lambda_1}\right)^{\frac{N}{2}-1}\frac{\partial \lambda_4}{\partial x}\right)}{1 + \left(\frac{\lambda_2}{\lambda_1}\right)^{\frac{N}{2}} + \left(\frac{\lambda_3}{\lambda_1}\right)^{\frac{N}{2}} + \left(\frac{\lambda_4}{\lambda_1}\right)^{\frac{N}{2}}} \xrightarrow{N\to\infty} \frac{x}{2\lambda_1}\frac{\partial \lambda_1}{\partial x} \qquad \text{S43.}$$

When N is odd and $m = (N-1)/2$, with Eq.S40, the binding density in Eq. S42 becomes:

$$\nu = \frac{x}{N}\frac{\partial}{\partial x}\ln\left(\lambda_1{}^m C_{11} + \lambda_2{}^m C_{22} + \lambda_3{}^m C_{33} + \lambda_4{}^m C_{44}\right)$$

$$= \frac{x}{N}\frac{\frac{N-1}{2}C_{11}\lambda_1{}^{\frac{N-3}{2}}\frac{\partial \lambda_1}{\partial x} + \frac{N-1}{2}C_{22}\lambda_2{}^{\frac{N-3}{2}}\frac{\partial \lambda_2}{\partial x} + \frac{N-1}{2}C_{33}\lambda_3{}^{\frac{N-3}{2}}\frac{\partial \lambda_3}{\partial x} + \frac{N-1}{2}C_{44}\lambda_4{}^{\frac{N-3}{2}}\frac{\partial \lambda_4}{\partial x}}{\lambda_1{}^{\frac{N-1}{2}}C_{11} + \lambda_2{}^{\frac{N-1}{2}}C_{22} + \lambda_3{}^{\frac{N-1}{2}}C_{33} + \lambda_4{}^{\frac{N-1}{2}}C_{44}}$$

$$+ \frac{x}{N}\frac{\lambda_1{}^{\frac{N-1}{2}}\frac{\partial C_{11}}{\partial x} + \lambda_2{}^{\frac{N-1}{2}}\frac{\partial C_{22}}{\partial x} + \lambda_3{}^{\frac{N-1}{2}}\frac{\partial C_{33}}{\partial x} + \lambda_4{}^{\frac{N-1}{2}}\frac{\partial C_{44}}{\partial x}}{\lambda_1{}^{\frac{N-1}{2}}C_{11} + \lambda_2{}^{\frac{N-1}{2}}C_{22} + \lambda_3{}^{\frac{N-1}{2}}C_{33} + \lambda_4{}^{\frac{N-1}{2}}C_{44}}$$

$$= \frac{x}{\lambda_1}\frac{N-1}{2N}\frac{C_{11}\frac{\partial \lambda_1}{\partial x} + C_{22}\left(\frac{\lambda_2}{\lambda_1}\right)^{\frac{N-3}{2}}\frac{\partial \lambda_2}{\partial x} + C_{33}\left(\frac{\lambda_3}{\lambda_1}\right)^{\frac{N-3}{2}}\frac{\partial \lambda_3}{\partial x} + C_{44}\left(\frac{\lambda_4}{\lambda_1}\right)^{\frac{N-3}{2}}\frac{\partial \lambda_4}{\partial x}}{C_{11} + C_{22}\left(\frac{\lambda_2}{\lambda_1}\right)^{\frac{N-1}{2}} + C_{33}\left(\frac{\lambda_3}{\lambda_1}\right)^{\frac{N-1}{2}} + C_{44}\left(\frac{\lambda_4}{\lambda_1}\right)^{\frac{N-1}{2}}}$$

$$+ \frac{x}{N}\frac{\frac{\partial C_{11}}{\partial x} + \left(\frac{\lambda_2}{\lambda_1}\right)^{\frac{N-1}{2}}\frac{\partial C_{22}}{\partial x} + \left(\frac{\lambda_3}{\lambda_1}\right)^{\frac{N-1}{2}}\frac{\partial C_{33}}{\partial x} + \left(\frac{\lambda_4}{\lambda_1}\right)^{\frac{N-1}{2}}\frac{\partial C_{44}}{\partial x}}{C_{11} + C_{22}\left(\frac{\lambda_2}{\lambda_1}\right)^{\frac{N-1}{2}} + C_{33}\left(\frac{\lambda_3}{\lambda_1}\right)^{\frac{N-1}{2}} + C_{44}\left(\frac{\lambda_4}{\lambda_1}\right)^{\frac{N-1}{2}}}$$

$$\xrightarrow{N\to\infty} \frac{x}{2\lambda_1}\frac{\partial \lambda_1}{\partial x} \qquad \text{S44}$$

In Eqs. S43 and S44, λ_1 is the eigenvalue with the largest absolute value of the transfer matrix P. Eqs. S43 and S44 show that regardless if the total lattice sites N is odd or even, as long as N is

very large (→∞), the binding density expression is the same and can be obtained by the largest absolute eigenvalue of the transfer matrix.

The physical meaning of λ_1 also becomes clear. According to Eqs. S39 and S40, the grand partition function can be written as:

$$\Xi = \begin{cases} \lambda_1{}^m \left(1 + \left(\frac{\lambda_2}{\lambda_1} \right)^m + \left(\frac{\lambda_3}{\lambda_1} \right)^m + \left(\frac{\lambda_4}{\lambda_1} \right)^m \right), & N \text{ is even} \\ \lambda_1{}^m \left(C_{11} + \left(\frac{\lambda_2}{\lambda_1} \right)^m C_{22} + \left(\frac{\lambda_3}{\lambda_1} \right)^m C_{33} + \left(\frac{\lambda_4}{\lambda_1} \right)^m C_{44} \right), & N \text{ is odd} \end{cases}$$
$$\xrightarrow{N\to\infty} \begin{cases} \lambda_1{}^m, & N \text{ is even} \\ \lambda_1{}^m C_{11}, & N \text{ is odd} \end{cases} \qquad \text{S45,}$$

indicating that when N is sufficiently large and end effects are negligible, λ_1 represents the contribution of each interaction unit (formed by 2 consecutive lattice sites, Eqs. S35 and S36, Table S1) to $\Xi(N)$, similar to the physical meaning of λ_1 in the exact solution by the sequence generating method (Eqs. 1-5 in the main text).

The equation for solving the eigenvalues of P is given by:

$$\left|P-\lambda\right| = \begin{vmatrix} 1-\lambda & 1 & 1 & 1 \\ x & x\omega_2-\lambda & x & x\omega_2 \\ x & x\omega_1 & x\omega_2-\lambda & x\omega_1\omega_2 \\ x^2\omega_1 & x^2\omega_1{}^2\omega_2 & x^2\omega_1\omega_2 & x^2\omega_1{}^2\omega_2{}^2-\lambda \end{vmatrix} = \left(1-\lambda\right) \begin{vmatrix} x\omega_2-\lambda & x & x\omega_2 \\ x\omega_1 & x\omega_2-\lambda & x\omega_1\omega_2 \\ x^2\omega_1{}^2\omega_2 & x^2\omega_1\omega_2 & x^2\omega_1\omega_2{}^2-\lambda \end{vmatrix}$$
$$- \begin{vmatrix} x & x & x\omega_2 \\ x & x\omega_2-\lambda & x\omega_1\omega_2 \\ x^2\omega_1 & x^2\omega_1\omega_2 & x^2\omega_1{}^2\omega_2{}^2-\lambda \end{vmatrix} + \begin{vmatrix} x & x\omega_2-\lambda & x\omega_2 \\ x & x\omega_1 & x\omega_1\omega_2 \\ x^2\omega & x^2\omega_1{}^2\omega_2 & x^2\omega_1{}^2\omega_2{}^2-\lambda \end{vmatrix} - \begin{vmatrix} x & x\omega_2-\lambda & x \\ x & x\omega_1 & x\omega_2-\lambda \\ x^2\omega_1 & x^2\omega_1{}^2\omega_2 & x^2\omega_1\omega_2 \end{vmatrix}$$
$$= \left(1-\lambda\right)\Big(\left(x\omega_2-\lambda\right)^2 \left(x^2\omega_1{}^2\omega_2{}^2-\lambda\right) + x^4\omega_1{}^2\omega_2{}^2 + x^4\omega_1{}^3\omega_2{}^2 - x^3\omega_1{}^2\omega_2{}^2\left(x\omega_2-\lambda\right)$$
$$-\left(x^2\omega_1{}^2\omega_2{}^2-\lambda\right)x^2\omega_1 - \left(x\omega_2-\lambda\right)x^3\omega_1{}^2\omega_2{}^2 \Big) - \left(x^2\omega_1{}^2\omega_2{}^2-\lambda\right)\left(x\omega_2-\lambda\right)x - x^4\omega_1{}^2\omega_2$$
$$-x^4\omega_1\omega_2{}^2 + \left(x\omega_2-\lambda\right)x^3\omega_1\omega_2 + \left(x^2\omega_1{}^2\omega_2{}^2-\lambda\right)x^2 + x^4\omega_1{}^2\omega_2{}^2 + \left(x^2\omega_1{}^2\omega_2{}^2-\lambda\right)x^2\omega_1$$
$$+\left(x\omega_2-\lambda\right)x^3\omega_1{}^2\omega_2 + x^4\omega_1{}^2\omega_2{}^2 - x^4\omega_1{}^2\omega_2 - \left(x^2\omega_1{}^2\omega_2{}^2-\lambda\right)\left(x\omega_2-\lambda\right)x - x^4\omega_1{}^3\omega_2{}^2$$
$$-x^4\omega_1{}^2\omega_2 - x^4\omega_1{}^2\omega_2 - \left(x\omega_2-\lambda\right)^2 x^2\omega_1 + x^4\omega_1{}^2 + \left(x\omega_2-\lambda\right)x^3\omega_1{}^2\omega_2 + \left(x\omega_2-\lambda\right)x^3\omega_1\omega_2$$

$$
\begin{aligned}
&= x^4\omega_1^{\,2} - 4x^4\omega_1^{\,2}\omega_2 + 6x^4\omega_1^{\,2}\omega_2^{\,2} - 4x^4\omega_1^{\,2}\omega_2^{\,3} + x^4\omega_1^{\,2}\omega_2^{\,4} \\
&\quad -x^2\lambda + 2x^2\omega_2\lambda - 2x^3\omega_1^{\,2}\omega_2\lambda - x^2\omega_2^{\,2}\lambda + 4x^3\omega_1^{\,2}\omega_2^{\,2}\lambda - x^4\omega_1^{\,2}\omega_2^{\,2}\lambda - 2x^3\omega_1^{\,2}\omega_2^{\,3}\lambda + 2x^4\omega_1^{\,2}\omega_2^{\,3}\lambda \\
&\quad -x^4\omega_1^{\,2}\omega_2^{\,4}\lambda - 2x\lambda^2 - 2x^2\omega_1\lambda^2 + 2x\omega_2\lambda^2 + x^2\omega_2^{\,2}\lambda^2 + x^2\omega_1^{\,2}\omega_2^{\,2}\lambda^2 - 2x^3\omega_1^{\,2}\omega_2^{\,2}\lambda^2 + 2x^3\omega_1^{\,2}\omega_2^{\,3}\lambda^2 \\
&\quad -\lambda^3 - x^2\omega_1^{\,2}\omega_2^{\,2}\lambda^3 - 2x\omega_2\lambda^3 + \lambda^4 \\
&= \lambda^4 - \left(1 + x^2\omega_1^{\,2}\omega_2^{\,2} + 2x\omega_2\right)\lambda^3 + x\left(2(\omega_2 - 1)\left(1 + x^2\omega_1^{\,2}\omega_2^{\,2}\right) + x\omega_2^{\,2} - 2x\omega_1 + x\omega_1^{\,2}\omega_2^{\,2}\right)\lambda^2 \\
&\quad -x^2(\omega_2 - 1)^2\left(1 + 2x\omega_1^{\,2}\omega_2 + x^2\omega_1^{\,2}\omega_2^{\,2}\right)\lambda + x^4\omega_1^{\,2}(\omega_2 - 1)^4 = 0 \qquad \text{S46}
\end{aligned}
$$

The roots λ_i of Eq. S46 are the eigenvalues of the transfer matrix *P*, and the largest absolute root is the one of interest for calculating the binding density ν (Eq. S43 or S44). Therefore, Eq S46 is the characteristic equation for binding density calculations.

We will prove later that the largest absolute root is positive and $\lambda_1 \geq 1$. Similar to the characteristic equation (Eq. 9 in the main text), derived from the sequence generating method, Eq. S46 is a 4th order polynomial and cannot be solved analytically, requiring a numeric procedure to be explored.

λ_1 is a function of *x* and to find its derivative respect to *x*, we take derivative of the both sides of Eq. S46 respect to *x*.

$$
\begin{aligned}
\frac{\partial f(x,\lambda)}{\partial x} &= 4x^3\omega_1^{\,2}(\omega_2 - 1)^4 - 2x(\omega_2 - 1)^2\left(1 + 2x\omega_1^{\,2}\omega_2 + x^2\omega_1^{\,2}\omega_2^{\,2}\right)\lambda \\
&+\left(2(\omega_2 - 1)\left(1 + x^2\omega_1^{\,2}\omega_2^{\,2}\right) + x\omega_2^{\,2} - 2x\omega_1 + x\omega_1^{\,2}\omega_2^{\,2}\right)\lambda^2 + x\left(4x\omega_1^{\,2}\omega_2^{\,2}(\omega_2 - 1) + \omega_2^{\,2} - 2\omega_1 + \omega_1^{\,2}\omega_2^{\,2}\right)\lambda^2 \\
&-2x^2\omega_1^{\,2}\omega_2(\omega_2 - 1)^2(1 + x\omega_2)\lambda - x^2(\omega_2 - 1)^2\left(1 + 2x\omega_1^{\,2}\omega_2 + x^2\omega_1^{\,2}\omega_2^{\,2}\right)\frac{\partial\lambda}{\partial x} \\
&+2x\left(2(\omega_2 - 1)\left(1 + x^2\omega_1^{\,2}\omega_2^{\,2}\right) + x\omega_2^{\,2} - 2x\omega_1 + x\omega_1^{\,2}\omega_2^{\,2}\right)\lambda\frac{\partial\lambda}{\partial x} \\
&-2\left(x\omega_1^{\,2}\omega_2^{\,2} + \omega_2\right)\lambda^3 - 3\left(1 + x^2\omega_1^{\,2}\omega_2^{\,2} + 2x\omega_2\right)\lambda^2\frac{\partial\lambda}{\partial x} + 4\lambda^3\frac{\partial\lambda}{\partial x} \\
&= 4x^3\omega_1^{\,2}(\omega_2 - 1)^4 - 2x(\omega_2 - 1)^2\left(1 + 3x\omega_1^{\,2}\omega_2 + 2x^2\omega_1^{\,2}\omega_2^{\,2}\right)\lambda \\
&+2\left((\omega_2 - 1)\left(1 + 3x^2\omega_1^{\,2}\omega_2^{\,2}\right) - 2x\omega_1 + x\omega_2^{\,2} + x\omega_1^{\,2}\omega_2^{\,2}\right)\lambda^2 - 2\omega_2\left(x\omega_1^{\,2}\omega_2 + 1\right)\lambda^3 \\
&-x^2(\omega_2 - 1)^2\left(1 + 2x\omega_1^{\,2}\omega_2 + x^2\omega_1^{\,2}\omega_2^{\,2}\right)\frac{\partial\lambda}{\partial x} + 2x\left(2(\omega_2 - 1)\left(1 + x^2\omega_1^{\,2}\omega_2^{\,2}\right) + x\omega_2^{\,2} - 2x\omega_1 + x\omega_1^{\,2}\omega_2^{\,2}\right)\lambda\frac{\partial\lambda}{\partial x} \\
&-3\left(1 + x^2\omega_1^{\,2}\omega_2^{\,2} + 2x\omega_2\right)\lambda^2\frac{\partial\lambda}{\partial x} + 4\lambda^3\frac{\partial\lambda}{\partial x} \\
&= 0
\end{aligned}
$$

The derivative of λ to x is:

$$\frac{\partial\lambda}{\partial x}=\frac{\begin{array}{l}4x^3\omega_1^{\,2}\left(\omega_2-1\right)^4-2x\left(\omega_2-1\right)^2\left(1+3x\omega_1^{\,2}\omega_2+2x^2\omega_1^{\,2}\omega_2^{\,2}\right)\lambda\\+2\left(\left(\omega_2-1\right)\left(1+3x^2\omega_1^{\,2}\omega_2^{\,2}\right)-2x\omega_1+x\omega_2^{\,2}+x\omega_1^{\,2}\omega_2^{\,2}\right)\lambda^2-2\omega_2\left(x\omega_1^{\,2}\omega_2+1\right)\lambda^3\end{array}}{\begin{array}{l}x^2\left(\omega_2-1\right)^2\left(1+2x\omega_1^{\,2}\omega_2+x^2\omega_1^{\,2}\omega_2^{\,2}\right)-2x\left(2\left(\omega_2-1\right)\left(1+x^2\omega_1^{\,2}\omega_2^{\,2}\right)+x\omega_2^{\,2}-2x\omega_1+x\omega_1^{\,2}\omega_2^{\,2}\right)\lambda\\+3\left(1+x^2\omega_1^{\,2}\omega_2^{\,2}+2x\omega_2\right)\lambda^2-4\lambda^3\end{array}}$$

S47

Substituting Eq. S47 into Eq. S43 or S44 yields the exact solution of the model for calculating binding density ν as function of x and λ_1, the largest root of Eq. S46 or largest eigenvalue of the transfer matrix, as in the following equation (Eq. S48):

$$\frac{\nu}{x}=\frac{1}{\lambda_1}\frac{\begin{array}{l}2x^3\omega_1^{\,2}\left(\omega_2-1\right)^4-x\left(\omega_2-1\right)^2\left(1+3x\omega_1^{\,2}\omega_2+2x^2\omega_1^{\,2}\omega_2^{\,2}\right)\lambda_1\\+\left(\left(\omega_2-1\right)\left(1+3x^2\omega_1^{\,2}\omega_2^{\,2}\right)-2x\omega_1+x\omega_2^{\,2}+x\omega_1^{\,2}\omega_2^{\,2}\right)\lambda_1^{\,2}-\omega_2\left(x\omega_1^{\,2}\omega_2+1\right)\lambda_1^{\,3}\end{array}}{\begin{array}{l}x^2\left(\omega_2-1\right)^2\left(1+2x\omega_1^{\,2}\omega_2+x^2\omega_1^{\,2}\omega_2^{\,2}\right)-2x\left(2\left(\omega_2-1\right)\left(1+x^2\omega_1^{\,2}\omega_2^{\,2}\right)-2x\omega_1+x\omega_2^{\,2}+x\omega_1^{\,2}\omega_2^{\,2}\right)\lambda_1\\+3\left(1+x^2\omega_1^{\,2}\omega_2^{\,2}+2x\omega_2\right)\lambda_1^{\,2}-4\lambda_1^{\,3}\end{array}}$$

S48

Similar to Eq. 11 in the main text, Eq. S48 is an exact solution of the double-stranded Ising lattice model that can be used for calculating the ligand binding density. To do that, the largest positive root of characteristic equation Eq. S46 or the largest eigenvalue of the transfer matrix *P* in Eq. S37 has to be numerically solved in the range $\lambda_1 \geq 1$ (see below). Simulated binding curves by Eqs. S46 and S48 from the transfer matrix method overlay those simulated with Eqs. 9 and 11 in the main text derived by the sequence generating method (Fig. S1), as expected.

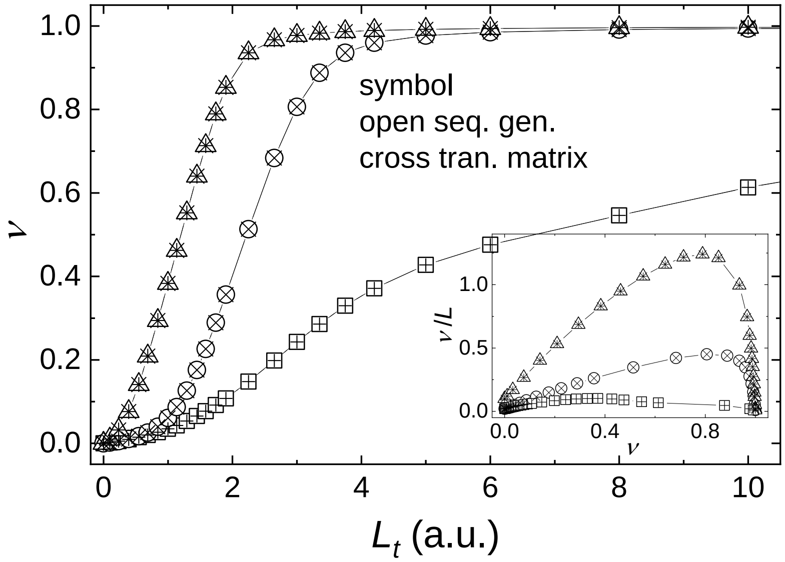

Figure S1. Simulations iterated according to the exact solutions by the sequence generating (open symbols) and by transfer matrix (cross symbols) methods for equilibrium ligand binding to the double-stranded Ising lattice model overlay. Inset: the same data plotted in Scatchard format (ν/L vs. ν). The parameters used in the simulations are K_d = 50, ω_1 = 0.5, ω_2 = 17, A_t = 1.5 (squares and right crosses), K_d = 50, ω_1 = 17, ω_2 = 2, A_t = 1.5 (circles and tilted crosses) and K_d = 10, ω_1 = 10, ω_2 = 2, A_t = 1.4 (triangles and stars).

8. *Proof that the root with the largest absolute value of characteristic equation Eq. S46 is* $\lambda_1 \geqslant 1$

Similar to the proof in Section 1 above, we let $f(\lambda)$ equal to the left side of Eq. S46 as a function of λ:

$$f\left(\lambda\right)=\lambda^4-\left(1+x^2\omega_1^{\,2}\omega_2^{\,2}+2x\omega_2\right)\lambda^3+x\left(2\left(\omega_2-1\right)\left(1+x^2\omega_1^{\,2}\omega_2^{\,2}\right)+x\omega_2^{\,2}-2x\omega_1+x\omega_1^{\,2}\omega_2^{\,2}\right)\lambda^2$$
$$-x^2\left(\omega_2-1\right)^2\left(1+2x\omega_1^{\,2}\omega_2+x^2\omega_1^{\,2}\omega_2^{\,2}\right)\lambda+x^4\omega_1^{\,2}\left(\omega_2-1\right)^4$$

S49.

At roots of Eq.S49, $f(\lambda)$ = 0 and $f(\lambda)$ changes its sign as λ crosses a root. $x = K_aL_f$ is proportional to free ligand concentration L_f and therefore $x \geq 0$. At $x = 0$, Eq. S49 (Eq. S46) becomes

$$f(\lambda) = \lambda^4 - \lambda^3 = (\lambda - 1)\lambda^3 = 0 \qquad \text{S50}$$

and the largest root is $\lambda_1 = 1$ (the only other root, a multiple (triplet) root, is $\lambda = 0$).

At $x > 0$ and $\omega_2 \neq 1$, when $\lambda \to \pm\infty$, $f(\lambda) \to \lambda^4 > 0$ and when $\lambda = 0$, $f(0) = \omega_1^2 x^4(\omega_2 - 1)^4 > 0$ (i.e. $f(\lambda)$ has the same sign at $\lambda = 0$ as $\lambda \to \pm\infty$), indicating $f(\lambda)$ has possibly 0, 2, or 4 positive roots and, similarly, possibly 0, 2, or 4 negative roots. The root with the largest absolute value is of interest, and it could be positive or negative. When λ from $\pm\infty$ simultaneously approaches 0, we compare $f(|\lambda|)$ and $f(-|\lambda|)$ values at any given absolute $|\lambda|$ value to see which reaches 0 first and its correspondent root λ is the root with the largest absolute value. When $|\lambda|$ is very large, both $f(|\lambda|)$ and $f(-|\lambda|)$ are positive since $f(\pm\infty) > 0$. Given that:

$$f(|\lambda|) - f(-|\lambda|) = -2\left(1 + x^2\omega_1^2\omega_2^2 + 2x\omega_2\right)|\lambda|^3 - 2x^2(\omega_2 - 1)^2\left(1 + 2x\omega_1^2\omega_2 + x^2\omega_1^2\omega_2^2\right)|\lambda| < 0 \qquad \text{S51}$$

(i.e., $f(|\lambda|) > 0$ is always smaller than $f(-|\lambda|) > 0$), $f(|\lambda|)$ reaches 0 faster and its root $|\lambda_1|$ is positive and has the largest absolute value, thereby proving that the root of Eq. S46 with the largest absolute value is positive. Similar to that of the proof in Section 1 above, since $x > 0$, $\lambda_1 > 0$, and $\nu > 0$, from Eq. S43 or S44, it must be

$$\frac{\partial \lambda_1}{\partial x} = \frac{2\nu\lambda_1}{x} > 0 \qquad \text{S52.}$$

This means that for $x > 0$, the physically meaningful largest root of Eq. S46, λ_1, monotonically increases with x. Therefore, the minimum value of $\lambda_1 = 1$ is at $x = 0$, and when $x > 0$, $\lambda_1 > 1$.

When $\omega_2 = 1$, characteristic equation Eq. S46 becomes

$$f(\lambda) = \left(\lambda^2 - \left(1 + 2x + x^2\omega_1^2\right)\lambda + x^2(\omega_1 - 1)^2\right)\lambda^2 = 0 \qquad \text{S53,}$$

and the larger root of Eq. S53 is

$$\lambda_1 = \lambda_+ = \frac{1}{2}\left(1 + 2x + x^2\omega_1^2 + \sqrt{\left(1 + 2x + x^2\omega_1^2\right)^2 - 4x^2(\omega_1 - 1)^2}\right)$$

$$=\frac{1}{2}\left(1+2x+x^2\omega_1^{\,2}+\left(x\omega_1+1\right)\sqrt{\left(4x+\left(x\omega_1-1\right)^2\right)}\right)$$

$$\geq\frac{1}{2}\left(1+2x+x^2\omega_1^{\,2}+\left(x\omega_1+1\right)\sqrt{\left(x\omega_1-1\right)^2}\right)$$

$$=\begin{cases}\frac{1}{2}\left(1+2x+x^2\omega_1^{\,2}+\left(x^2\omega_1^{\,2}-1\right)\right)=x+x^2\omega_1^{\,2}\geq 1, & \text{if} \quad x\omega_1\geq 1\\ \frac{1}{2}\left(1+2x+x^2\omega_1^{\,2}+\left(1-x^2\omega_1^{\,2}\right)\right)=1+x\geq 1, & \text{if} \quad x\omega_1<1\end{cases} \qquad \text{S54}$$

The above proves that in any case, the root of Eq. S46 with the largest absolute value is $\lambda_1 \geq 1$.

9. *Special cases of ligand equilibrium binding density and characteristic equation derived from the transfer matrix method*

When $x \to 0$, characteristic equation Eq. S46 becomes

$$f\left(x,\,\lambda\right)=\lambda^4-\left(1+x^2\omega_1^{\,2}\omega_2^{\,2}+2x\omega_2\right)\lambda^3+x\left(2\left(\omega_2-1\right)\left(1+x^2\omega_1^{\,2}\omega_2^{\,2}\right)+x\omega_2^{\,2}-2x\omega_1+x\omega_1^{\,2}\omega_2^{\,2}\right)\lambda^2$$
$$-x^2\left(\omega_2-1\right)^2\left(1+2x\omega_1^{\,2}\omega_2+x^2\omega_1^{\,2}\omega_2^{\,2}\right)\lambda+x^4\omega_1^{\,2}\left(\omega_2-1\right)^4$$
$$\xrightarrow{x\to 0}\lambda^4-\lambda^3=\lambda^3\left(\lambda-1\right)=0 \qquad \text{S55}$$

The largest root is $\lambda_1 = 1$, and the binding density equation (Eq. S48) becomes:

$$\frac{\nu}{x}=\frac{1}{\lambda_1}\frac{\begin{array}{c}2x^3\omega_1^{\,2}\left(\omega_2-1\right)^4-x\left(\omega_2-1\right)^2\left(1+3x\omega_1^{\,2}\omega_2+2x^2\omega_1^{\,2}\omega_2^{\,2}\right)\lambda_1\\+\left(\left(\omega_2-1\right)\left(1+3x^2\omega_1^{\,2}\omega_2^{\,2}\right)-2x\omega_1+x\omega_2^{\,2}+x\omega_1^{\,2}\omega_2^{\,2}\right)\lambda_1^{\,2}-\omega_2\left(x\omega_1^{\,2}\omega_2+1\right)\lambda_1^{\,3}\end{array}}{\begin{array}{c}x^2\left(\omega_2-1\right)^2\left(1+2x\omega_1^{\,2}\omega_2+x^2\omega_1^{\,2}\omega_2^{\,2}\right)-2x\left(2\left(\omega_2-1\right)\left(1+x^2\omega_1^{\,2}\omega_2^{\,2}\right)-2x\omega_1+x\omega_2^{\,2}+x\omega_1^{\,2}\omega_2^{\,2}\right)\lambda_1\\+3\left(1+x^2\omega_1^{\,2}\omega_2^{\,2}+2x\omega_2\right)\lambda_1^{\,2}-4\lambda_1^{\,3}\end{array}}$$
$$\xrightarrow{x\to 0}\frac{1}{\lambda_1}\frac{\left(\omega_2-1\right)\lambda_1^{\,2}-\omega_2\lambda_1^{\,3}}{3\lambda_1^{\,2}-4\lambda_1^{\,3}}=\frac{1}{\lambda_1}\frac{\omega_2-1-\omega_2\lambda_1}{3-4\lambda_1}\xrightarrow{\lambda=1}1 \qquad \text{S56}$$

(i.e., in the Scatchard plot, y-axis starts from $\frac{\nu}{L_f}=K_a$).

When $\omega_2 = 1$ (i.e. no longitudinal binding cooperativity), characteristic equation (Eq. S46) becomes Eq. S53, its largest root is expressed in Eq. S54, and the binding density function in Eq. S48 becomes:

$$
\begin{aligned}
\frac{\nu}{x} &= \frac{1}{\lambda_1}\frac{\left(-2x\omega_1 + x + x\omega_1^2\right)\lambda_1^2 - \left(x\omega_1^2 + 1\right)\lambda_1^3}{-2x\left(-2x\omega_1 + x + x\omega_1^2\right)\lambda_1 + 3\left(1 + x^2\omega_1^2 + 2x\right)\lambda_1^2 - 4\lambda_1^3} \\
&= \frac{x\left(\omega_1 - 1\right)^2 - \left(x\omega_1^2 + 1\right)\lambda_1}{-2x^2\left(\omega_1 - 1\right)^2 + 3\left(1 + 2x + x^2\omega_1^2\right)\lambda_1 - 4\lambda_1^2} \\
&= \frac{x\left(\omega_1 - 1\right)^2 - \left(x\omega_1^2 + 1\right)\lambda_1}{-2x^2\left(\omega_1 - 1\right)^2 - \left(1 + 2x + x^2\omega_1^2\right)\lambda_1 + 4\left(1 + 2x + x^2\omega_1^2\right)\lambda_1 - 4\lambda_1^2} \\
&= \frac{x\left(\omega_1 - 1\right)^2 - \left(x\omega_1^2 + 1\right)\lambda_1}{-2x^2\left(\omega_1 - 1\right)^2 - \left(1 + 2x + x^2\omega_1^2\right)\lambda_1 + 4x^2\left(\omega_1 - 1\right)^2} \\
&= \frac{x\left(\omega_1 - 1\right)^2 - \left(x\omega_1^2 + 1\right)\lambda_1}{2x^2\left(\omega_1 - 1\right)^2 - \left(1 + 2x + x^2\omega_1^2\right)\lambda_1}
\end{aligned}
\qquad \text{S57}
$$

The characteristic equation (Eq. S53) is used in the denominator at the last step of the derivation above. Substituting the largest root λ_1 in Eq. S54 yields:

$$
\begin{aligned}
\frac{\nu}{x} &= \frac{2x\left(\omega_1 - 1\right)^2 - \left(x\omega_1^2 + 1\right)\left(1 + 2x + x^2\omega_1^2 + \left(x\omega_1 + 1\right)\sqrt{\left(4x + \left(x\omega_1 - 1\right)^2\right)}\right)}{4x^2\left(\omega_1 - 1\right)^2 - \left(1 + 2x + x^2\omega_1^2\right)\left(1 + 2x + x^2\omega_1^2 + \left(x\omega_1 + 1\right)\sqrt{\left(4x + \left(x\omega_1 - 1\right)^2\right)}\right)} \\
&= \frac{\left(1 + 3x\omega_1 - x\omega_1^2 + x^2\omega_1^3\right) + \left(x\omega_1^2 + 1\right)\sqrt{\left(4x + \left(x\omega_1 - 1\right)^2\right)}}{\left(4x + \left(x\omega_1 - 1\right)^2\right)\left(1 + x\omega_1\right) + \left(1 + 2x + x^2\omega_1^2\right)\sqrt{\left(4x + \left(x\omega_1 - 1\right)^2\right)}} \\
&= \frac{1}{\sqrt{\left(4x + \left(x\omega_1 - 1\right)^2\right)}}\frac{\left(1 + 3x\omega_1 - x\omega_1^2 + x^2\omega_1^3\right) + \left(x\omega_1^2 + 1\right)\sqrt{\left(4x + \left(x\omega_1 - 1\right)^2\right)}}{\left(1 + 2x + x^2\omega_1^2\right) + \left(1 + x\omega_1\right)\sqrt{\left(4x + \left(x\omega_1 - 1\right)^2\right)}} \\
&\times \frac{\left(1 + 2x + x^2\omega_1^2\right) - \left(1 + x\omega_1\right)\sqrt{\left(4x + \left(x\omega_1 - 1\right)^2\right)}}{\left(1 + 2x + x^2\omega_1^2\right) - \left(1 + x\omega_1\right)\sqrt{\left(4x + \left(x\omega_1 - 1\right)^2\right)}}
\end{aligned}
$$

$$= \frac{1}{\sqrt{\left(4x+\left(x\omega_1-1\right)^2\right)}} \frac{\begin{array}{c}\left(1+3x\omega_1 - x\omega_1{}^2 + x^2\omega_1{}^3\right)\left(1+2x+x^2\omega_1{}^2\right) - \left(1+x\omega_1\right)\left(x\omega_1{}^2+1\right)\left(4x+\left(x\omega_1-1\right)^2\right) \\ +\left(\left(x\omega_1{}^2+1\right)\left(1+2x+x^2\omega_1{}^2\right) - \left(1+x\omega_1\right)\left(1+3x\omega_1 - x\omega_1{}^2 + x^2\omega_1{}^3\right)\right)\sqrt{\left(4x+\left(x\omega_1-1\right)^2\right)}\end{array}}{\left(1+2x+x^2\omega_1{}^2\right)^2 - \left(1+x\omega_1\right)^2\left(4x+\left(x\omega_1-1\right)^2\right)}$$

$$= \frac{1}{\sqrt{\left(4x+\left(x\omega_1-1\right)^2\right)}} \frac{2x\left(\omega_1-1\right)^2\left(x\omega_1-1\right) + 2x\left(\omega_1-1\right)^2\sqrt{\left(4x+\left(x\omega_1-1\right)^2\right)}}{4x^2\left(\omega_1-1\right)^2}$$

$$= \begin{cases} \dfrac{x\omega_1 - 1 + \sqrt{\left(4x+\left(x\omega_1-1\right)^2\right)}}{2x\sqrt{\left(4x+\left(x\omega_1-1\right)^2\right)}}, & \omega_1 \neq 1 \\ \dfrac{1}{x+1}, & \omega_1 = 1 \end{cases} \qquad \text{S58.}$$

Eq. S58 is an explicit expression of ν as function of x (i.e., L_f) and ω_1, and the density function in Eq. S58 shows that when there is no binding cooperativity ($\omega_1 = \omega_2 = 1$), the binding curve is hyperbolic, typical of equilibrium binding to a single or multiple, non-interacting binding sites (8).

When $\omega_1 \neq 1$, we derive Scatchard form of binding equation as follows: Eq. S58 can be re-written as:

$$x\omega_1 - 1 + \sqrt{\left(4x+\left(x\omega_1-1\right)^2\right)} = 2\nu\sqrt{\left(4x+\left(x\omega_1-1\right)^2\right)}.$$

Re-arranging terms:

$$x\omega_1 - 1 = \left(2\nu-1\right)\sqrt{\left(4x+\left(x\omega_1-1\right)^2\right)}$$

and squaring the both sides yields:

$$x^2\omega_1{}^2 - 2x\omega_1 + 1 = \left(2\nu-1\right)^2\left(4x+\left(x\omega_1-1\right)^2\right) = 4x\left(2\nu-1\right)^2 + \left(x^2\omega_1{}^2 - 2x\omega_1 + 1\right)\left(2\nu-1\right)^2,$$

which can be rearranged to:

$$\nu(1-\nu)x^2\omega_1^{\ 2} - x\left(2\omega_1\nu(1-\nu)+(2\nu-1)^2\right)+\nu(1-\nu)=0 \qquad \text{S59.}$$

Solving Eq. S59 for x yields 2 solutions:

$$x=\frac{2\nu(1-\nu)(\omega_1-2)+1\pm\sqrt{\left(2\nu(1-\nu)(\omega_1-2)+1\right)^2-4\omega_1^{\ 2}\nu^2(1-\nu)^2}}{2\nu(1-\nu)\omega_1^{\ 2}}. \qquad \text{S60}$$

When $\nu\to 0$, $x_+\to\infty$, therefore, only x_- is physically meaningful. Using x_- in Scatchard form

$$\begin{aligned}\frac{\nu}{x}&=\frac{2\nu^2\omega_1^{\ 2}(1-\nu)}{2\nu(1-\nu)(\omega_1-2)+1-\sqrt{\left(2\nu(1-\nu)(\omega_1-2)+1\right)^2-4\omega_1^{\ 2}\nu^2(1-\nu)^2}}\\
&=2\nu^2\omega_1^{\ 2}(1-\nu)\frac{2\nu(1-\nu)(\omega_1-2)+1+\sqrt{\left(2\nu(1-\nu)(\omega_1-2)+1\right)^2-4\omega_1^{\ 2}\nu^2(1-\nu)^2}}{4\omega_1^{\ 2}\nu^2(1-\nu)^2}\\
&=\frac{2\nu(1-\nu)(\omega_1-2)+1+\sqrt{\left(2\nu(1-\nu)(\omega_1-2)+1\right)^2-4\omega_1^{\ 2}\nu^2(1-\nu)^2}}{2(1-\nu)}\\
&=\frac{2\nu(1-\nu)(\omega_1-2)+1+\sqrt{(1-2\nu)^2\left(1+4\nu(\omega_1-1)(1-\nu)\right)}}{2(1-\nu)}\\
&=\frac{(1-2\nu)^2+2\nu(1-\nu)\omega_1+\sqrt{(1-2\nu)^2\left((1-2\nu)^2+4\nu(1-\nu)\omega_1\right)}}{2(1-\nu)}\end{aligned} \qquad \text{S61}$$

Eq. S61 is the Scatchard binding equation for cooperative ligand binding to a 1-D lattice, previously reported by several groups (2, 3) and derived in this work by the sequence generating method (Eq. S13 above).

10. *Using the binding density equation Eq. S48 with the largest root of Eq. S46 for binding simulation and data analysis.*

For simulation and analysis of a binding curve as ν vs. L_f, the free ligand concentration, it is straight forward to use Eqs. S46 and S48, similar to that from the sequence generating method described in the main text. However, in experimental data, free ligand concentration is usually unknown, but the total ligand concentration is known instead. Therefore, the mass conservation of the total ligand concentration L_t has to be satisfied by free ligand $L_f = x\ K_d$ (K_d dissociation binding constant), and total lattice binding sites A_t,:

$$xK_d + vA_t = L_t \qquad \text{S62}$$

To solve x and ν using Eqs, S46 and $48 under constraints of Eq. S62, it is convenient to define an x and λ_1, the largest root of Eq. S46 (omit subscript "1" from now on for convenience), dependent function $f(x, \lambda_1)$ and its derivative with respect to x :

$$f(x,\lambda) = xK_d + vA_t - L_t \qquad \text{S63}$$

$$\frac{df}{dx} = K_d + A_t \frac{dv(x,\lambda)}{dx} = K_d + A_t \left(\frac{\partial v(x,\lambda)}{\partial x} + \frac{\partial v(x,\lambda)}{\partial \lambda} \frac{d\lambda}{dx} \right) = K_d + A_t \left(\frac{\partial v(x,\lambda)}{\partial x} + \frac{2\lambda v}{x} \frac{\partial v(x,\lambda)}{\partial \lambda} \right)$$
S64

Where $\frac{d\lambda}{dx} = \frac{2\lambda v}{x}$ (Eq. S43 or S44) is used. For the numerical approach to solve $f(x, \lambda) = 0$ with Eqs. S46 and S48. The following equations can be used.

$$v(x,\lambda) = \frac{x}{\lambda} \frac{N(x,\lambda)}{D(x,\lambda)} \qquad \text{S65}$$

$$\begin{aligned} N(x,\lambda) &= 2x^3\omega_1^2(\omega_2-1)^4 - x(\omega_2-1)^2\left(1+3x\omega_1^2\omega_2+2x^2\omega_1^2\omega_2^2\right)\lambda \\ &+\left((\omega_2-1)\left(1+3x^2\omega_1^2\omega_2^2\right)+x\omega_2^2-2x\omega_1+x\omega_1^2\omega_2^2\right)\lambda^2 - \omega_2\left(x\omega_1^2\omega_2+1\right)\lambda^3 \end{aligned} \qquad \text{S66}$$

$$D(x,\lambda) = x^2(\omega_2 - 1)^2\left(1 + 2x\omega_1{}^2\omega_2 + x^2\omega_1{}^2\omega_2{}^2\right)$$
$$-2x\left(2(\omega_2 - 1)\left(1 + x^2\omega_1{}^2\omega_2{}^2\right) + x\omega_2{}^2 - 2x\omega_1 + x\omega_1{}^2\omega_2{}^2\right)\lambda + 3\left(1 + x^2\omega_1{}^2\omega_2{}^2 + 2x\omega_2\right)\lambda^2 - 4\lambda^3 \qquad \text{S67}$$

$$\left.\frac{\partial v(x,\lambda)}{\partial x}\right|_\lambda = \frac{v(x,\lambda)}{x} + \frac{x}{\lambda}\frac{D(x,\lambda)\left.\frac{\partial N(x,\lambda)}{\partial x}\right|_\lambda - N(x,\lambda)\left.\frac{\partial D(x,\lambda)}{\partial x}\right|_\lambda}{D(x,\lambda)^2} \qquad \text{S68}$$

$$\left.\frac{\partial v(x,\lambda)}{\partial \lambda}\right|_x = -\frac{v(x,\lambda)}{\lambda} + \frac{x}{\lambda}\frac{D(x,\lambda)\left.\frac{\partial N(x,\lambda)}{\partial \lambda}\right|_x - N(x,\lambda)\left.\frac{\partial D(x,\lambda)}{\partial \lambda}\right|_x}{D(x,\lambda)^2} \qquad \text{S69}$$

$$\frac{\partial N(x,\lambda)}{\partial x} = 6x^2\omega_1{}^2(\omega_2 - 1)^4 - (\omega_2 - 1)^2\left(1 + 3x\omega_1{}^2\omega_2 + 2x^2\omega_1{}^2\omega_2{}^2\right)\lambda$$
$$-x\omega_1{}^2\omega_2(\omega_2 - 1)^2(3 + 4x\omega_2)\lambda + \left(6x\omega_1{}^2\omega_2{}^2(\omega_2 - 1) + \omega_2{}^2 - 2\omega_1 + \omega_1{}^2\omega_2{}^2\right)\lambda^2 - \omega_1{}^2\omega_2{}^2\lambda^3 \qquad \text{S70}$$

$$\frac{\partial D(x,\lambda)}{\partial x} = 2x(\omega_2 - 1)^2\left(1 + 2x\omega_1{}^2\omega_2 + x^2\omega_1{}^2\omega_2{}^2\right) + 2x^2\omega_1{}^2\omega_2(\omega_2 - 1)^2(1 + x\omega_2)$$
$$-2\left(2(\omega_2 - 1)\left(1 + x^2\omega_1{}^2\omega_2{}^2\right) + x\omega_2{}^2 - 2x\omega_1 + x\omega_1{}^2\omega_2{}^2\right)\lambda \qquad \text{S71}$$
$$-2x\left(4x\omega_1{}^2\omega_2{}^2(\omega_2 - 1) + \omega_2{}^2 - 2\omega_1 + \omega_1{}^2\omega_2{}^2\right)\lambda + 6\omega_2\left(x\omega_1{}^2\omega_2 + 1\right)\lambda^2$$

$$\left.\frac{\partial N(x,\lambda)}{\partial \lambda}\right|_x = -x(\omega_2 - 1)^2\left(1 + 3x\omega_1{}^2\omega_2 + 2x^2\omega_1{}^2\omega_2{}^2\right)$$
$$+2\left((\omega_2 - 1)\left(1 + 3x^2\omega_1{}^2\omega_2{}^2\right) + x\omega_2{}^2 - 2x\omega_1 + x\omega_1{}^2\omega_2{}^2\right)\lambda - 3\omega_2\left(x\omega_1{}^2\omega_2 + 1\right)\lambda^2 \qquad \text{S72}$$

$$\left.\frac{\partial D(x,\lambda)}{\partial \lambda}\right|_x = -2x\left(2(\omega_2 - 1)\left(1 + x^2\omega_1{}^2\omega_2{}^2\right) + x\omega_2{}^2 - 2x\omega_1 + x\omega_1{}^2\omega_2{}^2\right)$$
$$+6\left(1 + x^2\omega_1{}^2\omega_2{}^2 + 2x\omega_2\right)\lambda - 12\lambda^2 \qquad \text{S73,}$$

which can also be expressed in a logarithmic form:

$$f(x,\lambda) = \ln(Lt - xK_d) - \ln(vA_t) \quad \text{S74}$$

$$\begin{aligned}
\frac{df}{dx} &= \frac{d}{dx}\ln(L_t - xK_d) - \frac{d}{dx}\ln(vA_t) \\
&= \frac{-K_d}{L_t - xK_d} - \frac{d}{dx}(\ln x + \ln N - \ln\lambda - \ln D) \\
&= \frac{-1}{\frac{L_t}{K_d} - x} - \left(\frac{1}{x} + \frac{1}{N}\frac{dN}{dx} - \frac{1}{\lambda}\frac{d\lambda}{dx} - \frac{1}{D}\frac{dD}{dx}\right) \\
&= \frac{-1}{\frac{L_t}{K_d} - x} - \frac{1}{x} - \frac{\frac{\partial N}{\partial x} + \frac{\partial N}{\partial\lambda}\frac{\partial\lambda}{\partial x}}{N} + \frac{\frac{d\lambda}{dx}}{\lambda} + \frac{\frac{\partial D}{\partial x} + \frac{\partial D}{\partial\lambda}\frac{\partial\lambda}{\partial x}}{D} \\
&= \frac{-1}{\frac{L_t}{K_d} - x} - \frac{1}{x} - \frac{\frac{\partial N}{\partial x} + \frac{2\lambda v}{x}\frac{\partial N}{\partial\lambda}}{N} + \frac{2v}{x} + \frac{\frac{\partial D}{\partial x} + \frac{2\lambda v}{x}\frac{\partial\lambda}{\partial x}}{D}
\end{aligned} \quad \text{S75}$$

In this case, a numerical procedure has to be used to solve $f(x, \lambda_1) = 0$ in Eq. S74.

11. *Monte Carlo (MC) simulations of ligand binding kinetics to our double stranded lattice*

The methods used for the simulations were based on published MC procedures (9, 10), following Epstein's algorithm (9) and the multi-step binding kinetics treatment used by De La Cruz and Sept (11). The conditions for the MC simulations are described in the Methods section of the main text. Since our linearized double stranded model has additional cooperativities from either one or both second nearest neighbors, the maximum forward/binding (q_f) and reverse/dissociation (q_r) rate constants were modified accordingly from Epstein's (9) to:

$$q_f = k_f q_{1f} q_{2f}, \qquad q_r = k_r q_{1r} q_{2r} \quad \text{S76}$$

where

$$q_{1f,1r,2f,2r} = \begin{cases} \omega_{1f,1r,2f,2r}{}^2, & \text{if } \omega_{1f,1r,2f,2r} > 1 \\ 1, & \text{if } \omega_{1f,1r,2f,2r} \le 1 \end{cases} \qquad \text{S77}$$

In Eqs. S76 and S77, k_f and k_r are the forward (association) and reverse (dissociation) rate constants, and the two pairs of cooperativities: ω_{1f} ω_{1r} and ω_{2f} and ω_{2r} are the kinetic contributions of ω_1 and ω_2 to the rate constants originating from the occupied (first) nearest neighbors and second nearest neighbors, respectively. The relations of those rate constants with the equilibrium binding are $K_d = k_r/k_f$, $\omega_1 = \omega_{1f}/\omega_{1r}$, and $\omega_2 = \omega_{2f}/\omega_{2r}$ (12).

The probability thresholds for an attempt at association (P_f) or dissociation (P_r) to succeed are also modified accordingly from Epstein's (9) to:

$$P_f = P_{1f}P_{2f}, \quad P_r = P_{1r}P_{2r} \qquad \text{S78}$$

where P_{1f} and P_{1r} are the probability thresholds for binding and dissociation related to the nearest neighbors' occupancies and effects, the same as Epstein's, while P_{2f} and P_{2r} have the similar definitions but are for the second nearest neighbors, i.e.:

$$P_{1f,1r,2f,2r} = \begin{cases} \dfrac{1}{\omega_{1f,1r,2f,2r}{}^2}, & \text{no neighbor} \\ \dfrac{1}{\omega_{1f,1r,2f,2r}}, & \text{1 neighbor} \\ 1, & \text{2 neighbors} \end{cases}, \quad \omega_{1f,1r,2f,2r} > 1 \qquad \text{S79.}$$

$$P_{1f,1r,2f,2r} = \begin{cases} 1, & \text{no neighbor} \\ \omega_{1f,1r,2f,2r}, & \text{1 neighbor} \\ \omega_{1f,1r,2f,2r}{}^2, & \text{2 neighbors} \end{cases}, \quad \omega_{1f,1r,2f,2r} \le 1$$

If the time step or/and the total simulation time is/are not specified, the simulation time step Δt will be automatically set to 0.1/(the faster one of the maximum binding and dissociation $q_f L_f$ and q_r,), so that there is enough resolution to result the fast process, or/and the total simulation time for binding to reach equilibrium will be automatically set to 1/(the slower one of the minimum/slow binding and dissociation, which is opposite to their maximum values) to ensure simulations reach equilibrium. The Matlab *m-file* for this simulation is included as part of the *SI*.

One of the procedures in the Epstein algorithm involves permutation of all associations first followed by all dissociation reactions (9). We modified the event queue at each time step so that association and dissociation events were random rather than ordered. This modification eliminates the time step size problem (Fig. S2A) with the "all association before all dissociation" algorithm. Specifically, it eliminates the apparent loss of ligand binding at large time step sizes (Fig. S2B).

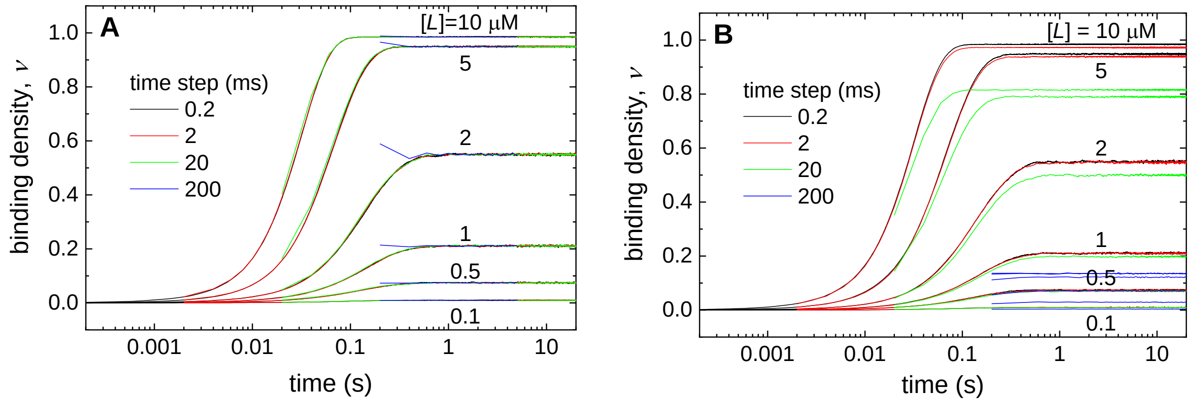

Figure S2: "Random association and dissociation" (A) vs. "all association before all dissociation" (B). Time courses for ligand binding to 1-D Ising lattice model were simulated by a Monte Carlo method (9) with association rate constant $k_+ = 1$, dissociation rate constant $k_- = 10$, kinetic cooperativity parameters $\omega_+ = 10$ for cooperativity contributions to k_+ and $\omega_- = 1$ for contributions to k_-, where $\omega = \omega_+/\omega_-$, [lattice sites] = 1.8 and the indicated ligand concentrations.

12. *ω_1 and ω_2 exchange symmetry in the double-stranded lattice model is non-continuous.*

The observed exchange symmetry between the two cooperativities ω_1 *and* ω_2 (Figure 3) raises the possibility that a continuous symmetry may exist such that similar binding curves are obtained when ω_1 and ω_2 change magnitude in opposite direction in a continuous manner. A plot of the mean square error (proportional to chi squared) of an experimental data set and simulated binding curves with ω_1 and ω_2 changing in opposite directions and their sums constrained (Fig. S3) shows two distinct fitting minima, consistent with an exchange symmetry only between two unique values of ω_1 and ω_2, thereby demonstrating that a continuous symmetry does not exist in the fit parameter space of the data.

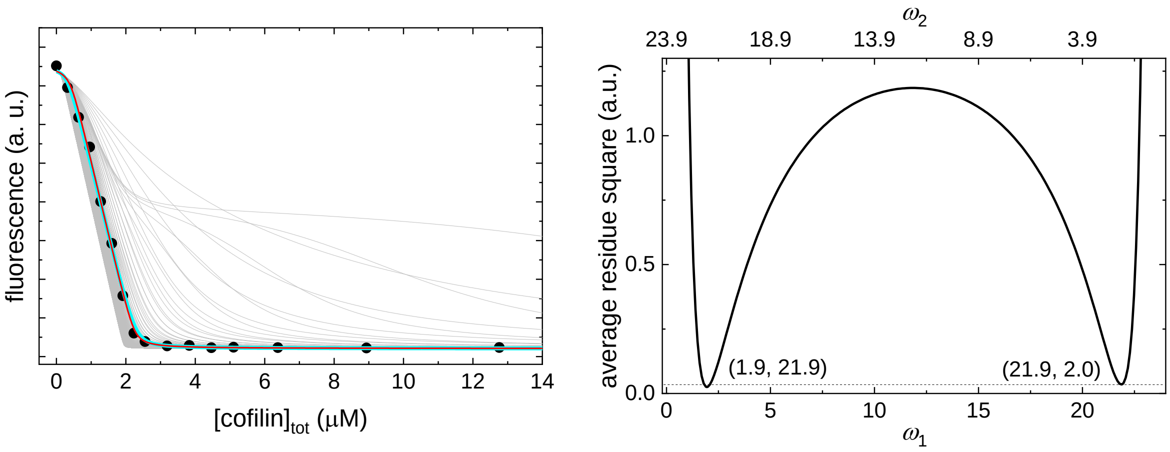

Figure S3. The exchange symmetry is the only symmetry between the two binding cooperativities ω_1 and ω_2, and the other value pairs of two parameters generate different binding curves. Left panel: an individual experimental titration curve data set (filled black circles) of cofilin binding to actin filaments overlaid with the best fit of the data to the double-stranded lattice model (red line; K_d = 18.4 μM, ω_1 = 1.9 and ω_2 = 21.9), a simulated binding curve with ω_1 and ω_2 values exchanged (cyan line; K_d = 18.4 μM, ω_1 = 21.9 and ω_2 = 1.9), and other combinations of ω_1 and ω_2 value pairs, changed in opposite directions with their sum constrained (light grey lines; K_d = 18.4 μM, $\omega_1 + \omega_2$ = 23.9). The experimental conditions are described in the Methods section of the main text, with [actin] = 1.8 μM and [cofilin] as indicated along the *x*-axis. The fitting and simulations were done using Eqs. 9 and 11; the solution obtained with the sequence generating method are described in detail in the Methods section of the main text. Right panel: ω_1- and ω_2-dependence of the average squared residual. The average squared residuals (Eq. S80) of the *y*-difference between each data point and the corresponding simulated value (as plotted in the left panel) yields the maximal likelihood (i.e., the minimum) for the simulated data to match the experimental data. The ω_1 and ω_2 values for the two best fits (left and right minima, respectively) are labeled in the plot. The horizontal dashed line coincides with the *y*-value of the right minimum (ω_1 = 21.9 and ω_2 = 1.9) and shows that it is slightly higher than that of the left minimum (ω_1 = 1.9 and ω_2 = 21.9), indicating the ω_1 and ω_2 exchange symmetry is not exact.

$$\text{average squared residual} = \frac{\sum_{i=1}^{n}\left(y\left(x_i\right)-y_i\right)^2}{n} \qquad \text{S80.}$$

13. *Attached computer program files*

1. equil_2D_coop_binding_sequence_generating.m

A Matlab *m*-file (source code) for simulating equilibrium ligand binding to double stranded lattice model with the nearest neighbor cooperativities by the solution solved with the sequence generating method.

2. coop_bind_to_double_strend_lattice_sequnce_generating.FDF
   Software Origin (originlab.com) fitting program for fitting data to equilibrium ligand binding to double stranded lattice with nearest neighbor cooperatives, written in Origin-C, a combination of *C* and *C*++ programming language, and compiled including environment parameter settings. The program can be easily imported and used with Origin.

   The file coop_bind_to_double_strend_lattice_sequnce_generating.c is the source code of the fitting program in plain text format.

3. MC_coop_ligand_bind_double_stranded.m
   A Monte Carlo algorithm written in Matlab scripts (*m*-file; source code) for simulation of ligand binding to a double stranded lattice with longitudinal and lateral, nearest neighbor cooperativities.

References

1. S. Lifson, Partition Functions of Linear-Chain Molecules. *J Chem Phys* **40**, 3705-3710 (1964).
2. T. Tsuchiya, A. Szabo, Cooperative binding of n-mers with steric hindrance to finite and infinite one-dimensional lattices. *Biopolymers* **21**, 979-994 (1982).
3. J. McGhee, P. Von Hippel, Theoretical aspects of DNA-protein interactions: Co-operative and non-co-operative binding of large ligands to a one-dimensional homogeneous lattice. *Journal of molecular biology* **86**, 469-489 (1974).
4. S. Kowalczykowski *et al.*, Cooperative and noncooperative binding of protein ligands to nucleic acid lattices: Experimental approaches to the determination of thermodynamic parameters. *Biochemistry* **25**, 1226-1240 (1986).
5. D. M. Crothers, Calculation of binding isotherms for heterogeneous polymers. *Biopolymers* **6**, 575-584 (1968).
6. G. Schwarz, General theoretical approach to the thermodynamic and kinetic properties of cooperative intramolecular transformations of linear biopolymers. *Biopolymers* **6**, 873-897 (1968).
7. G. Schwarz, Cooperative binding to linear biopolymers. 1. Fundamental static and dynamic properties. *Eur J Biochem* **12**, 442-453 (1970).
8. J. Wyman, S. J. Gill, *Binding and linkage : functional chemistry of biological macromolecules* (University Science Books, Mill Valley, Calif., 1990), pp. xiii, 330 p.
9. I. R. Epstein, Kinetics of nucleic acid-large ligand interactions: exact Monte Carlo treatment and limiting cases of reversible binding. *Biopolymers* **18**, 2037-2050 (1979).
10. D. T. Gillespie, Exact Stochastic Simulation of Coupled Chemical-Reactions. *Journal of Physical Chemistry* **81**, 2340-2361 (1977).
11. E. M. De La Cruz, D. Sept, The kinetics of cooperative cofilin binding reveals two states of the cofilin-actin filament. *Biophysical journal* **98**, 1893-1901 (2010).
12. W. Cao, J. P. Goodarzi, E. M. De La Cruz, Energetics and kinetics of cooperative cofilin-actin filament interactions. *Journal of molecular biology* **361**, 257-267 (2006).
